# Atomically Dispersed Platinum Modulated by Sulfide as an Efficient Electrocatalyst for Hydrogen Evolution Reaction

**DOI:** 10.1002/advs.202100347

**Published:** 2021-05-13

**Authors:** Kai Ling Zhou, Chang Bao Han, Zelin Wang, Xiaoxing Ke, Changhao Wang, Yuhong Jin, Qianqian Zhang, Jingbing Liu, Hao Wang, Hui Yan

**Affiliations:** ^1^ Faculty of Materials and Manufacturing Key Laboratory of Advanced Functional Materials Education Ministry of China Beijing University of Technology Beijing 100124 P. R. China

**Keywords:** architectural nanostructure engineering, hydrogen evolution reaction (HER), metal‐support interaction, single‐atom catalysts (SACs), sulfides

## Abstract

Catalytically active metals atomically dispersed on supports presents the ultimate atom utilization efficiency and cost‐effective pathway for electrocatalyst design. Optimizing the coordination nature of metal atoms represents the advanced strategy for enhancing the catalytic activity and the selectivity of single‐atom catalysts (SACs). Here, we designed a transition‐metal based sulfide‐Ni_3_S_2_ with abundant exposed Ni vacancies created by the interaction between chloride ions and the functional groups on the surface of Ni3S2 for the anchoring of atomically dispersed Pt (Pt_SA_‐Ni_3_S_2_). The theoretical calculation reveals that unique Pt‐Ni_3_S_2_ support interaction increases the *d* orbital electron occupation at the Fermi level and leads to a shift‐down of the *d* ‐band center, which energetically enhances H_2_O adsorption and provides the optimum H binding sites. Introducing Pt into Ni position in Ni_3_S_2_ system can efficiently enhance electronic field distribution and construct a metallic‐state feature on the Pt sites by the orbital hybridization between S‐3p and Pt‐5d for improved reaction kinetics. Finally, the fabricated Pt_SA_‐Ni_3_S_2_ SAC is supported by Ag nanowires network to construct a seamless conductive three‐dimensional (3D) nanostructure (Pt_SA_‐Ni_3_S_2_@Ag NWs), and the developed catalyst shows an extremely great mass activity of 7.6 A mg^−1^ with 27‐time higher than the commercial Pt/C HER catalyst.

## Graphical abstract

A transition‐metal sulfide‐Ni_3_S_2_, with abundant exposed Ni vacancies in the outside layers, to anchor and modulate atomically dispersed Pt as the durable catalyst (Pt_SA_‐Ni_3_S_2_) for efficiently electrocatalytic hydrogen evolution.

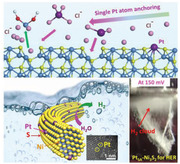

## 1 Introduction

The noble‐metals based catalysts play significant roles in renewable energy utilization, storage, and conversion,^[^ ^1^, ^2^ ^]^ and have been widely used to drive the various catalytic reactions including nitrogen reduction reaction,^[^ ^3^, ^4^ ^]^ hydrogen evolution reaction (HER),^[^ ^5^, ^6^ ^]^ oxygen evolution reaction,^[^ ^7^, ^8^ ^]^ and the oxygen reduction reaction.^[^ ^9^, ^10^ ^]^ However, the high cost caused by scarcity hinders the widespread application of these catalysts. Single‐atom catalysts (SACs), with atomically distributed noble‐metal on supports equipped with extremely high specific activity and significantly large atom utilization efficiency, have attracted considerable attention.^[^ ^11^ ^]^ The electrochemical activity of single‐atom sites highly depends on the chemical and physical properties of the SACs, and specifically, the microenvironments of the single atom, including geometric construction and electronic coordination are extremely critical in determinating the performances of the catalysts.^[^ ^12^, ^13^ ^]^ Therefore, exploring and understanding the structure‐activity relationship of the single‐atom in SACs is imperative from the aspects of materials synthesis and reaction mechanism.

For the SACs, the strong interaction between the metal atoms and support is required to avoid the formation of aggregated clusters and particles by considering the stability.^[^ ^14^, ^15^ ^]^ However, the strong metal‐support interaction will result in a large charge loss of metal atoms, and gift a cation‐like positive electrons states to the metal atom.^[^ ^16^, ^17^ ^]^ These high‐valence metal atoms are more favorable for electro‐oxidation reactions rather than electroreduction processes due to the less *d* orbital electrons of SACs involved in the reaction.^[^ ^13^ ^]^ Recently, the metal atoms with low‐coordinated electrons transfer and the close‐to‐zero valence state resembling a metallic‐state atom were designed by anchoring Pt on the specific site of N‐C framework and exhibited a superior activity for electroreduction reactions.^[^ ^18^ ^]^ Whereas, the application of such metallic‐state atoms is embarrassed due to the unaccessible fabrication approach and unsatisfied durability. Further, the local electron distribution around metal sites and *d*‐band position of SACs is sensitive to the electron interaction between the metal atom and support,^[^ ^16^, ^17^ ^]^ and especially, the locally enhanced electron density on the single‐metal sites induced by appropriate metal‐support interaction has been expected to efficiently accelerate the catalytic reaction kinetics toward HER.^[^ ^19^, ^20^ ^]^ Nevertheless the development of such unique electronic field distribution feature in SACs are extremely sluggish due to the lack of suitable support. Therefore, it is challenging but significant to fabricate the metallic‐state single atoms in the catalyst with the optimized *d* orbital structure and locally enhanced electronic field distribution by increase the local electron density on the metal atoms for satisfactory HER by considering the stability. Apart from the electronic structure factors, the catalytic activity of SACs is also extremely limited by low exposure efficiency of metal atoms sites and inefficient electrons supply ability due to the introduction of insulating binder (e.g., Nafion and polytetrafluoroethylene) in electrode framework,^[^ ^13^, ^14^, ^19^, ^21^, ^22^ ^]^ leading to the inefficient conversion of the adsorbed hydrogen (M‐H_ads_) to H_2_ in the Heyrovsky or Tafel step.^[^ ^23^, ^24^, ^25^, ^26^, ^27^ ^]^ Interesting, architectural nanostructure engineering, in the non‐noble metal catalysts field, provides an appealing platform to realize a seamlessly conductive system by integrating active materials on current collectors.^[^ ^28^, ^29^ ^]^ Inspired by the above, the integration of electronic structure adjustment strategy and architectural nanostructure engineering may provide some new avenues and insights for the design of highly efficient SACs.

Here, by combining theoretical simulation techniques, we design a transition‐metal sulfide‐Ni_3_S_2_, with abundant exposed Ni vacancies created by the interaction between chloride ions and the functional groups on the surface of Ni_3_S_2_, to anchor atomically dispersed Pt for efficiently electrocatalytic hydrogen evolution, Specifically, the Pt atoms undergo a redistribution in the local electronic structure after coordinating with Ni_3_S_2_ support and lead to a locally enhanced electronic field distribution by increasing the electron density on the top of Pt sites. Meanwhile, metallic‐state single Pt atoms are obtained by the orbital hybridization between S‐3*p* and Pt‐5*d* after introducing Pt into Ni position in the Ni_3_S_2_ system for improved reaction kinetics. Moreover, Pt‐Ni_3_S_2_ support interaction increases the *d* orbital electron occupation at the Fermi level and leads to a shift‐down of the *d*‐band center, which energetically enhances H_2_O adsorption and provides the optimum H binding sites. By considering the superiority of architectural nanostructure, Ni_3_S_2_ toiled Pt single atom is integrated on Ag nanowires network (Pt_SA_‐Ni_3_S_2_@Ag NWs) to form a seamlessly conductive nanostructure for the more facile generation and release of H_2_. As such, the designed Pt_SA_‐Ni_3_S_2_@Ag NWs demonstrate outstanding HER performance with 27‐fold higher mass activity than the commercial Pt/C catalyst and long‐term durability with 5000 cycles or 30 h.

## 2 Results and Discussion

**Figure 1:**
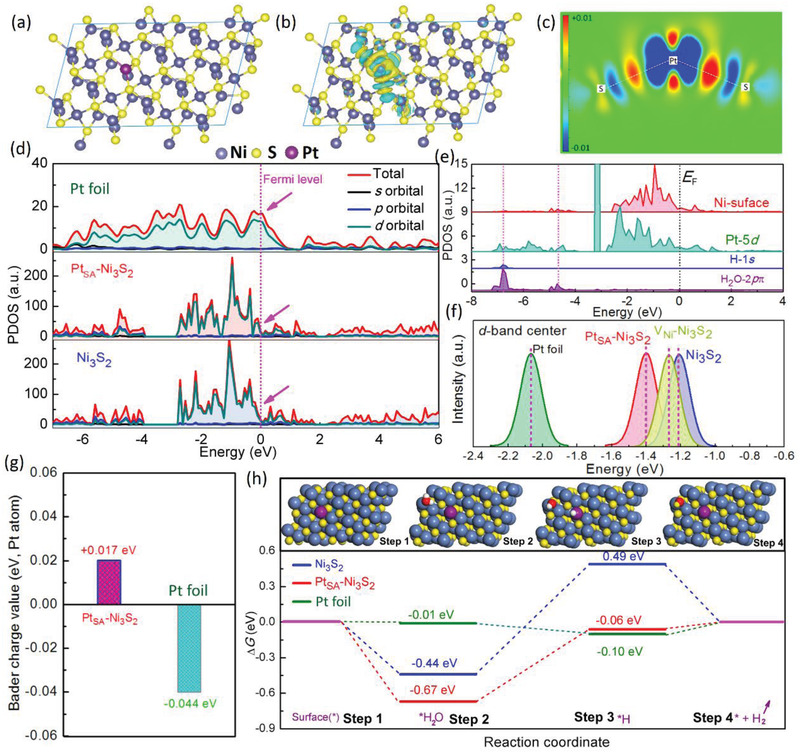
Theoretical investigations. a) Top view of the computational model of Pt_SA_–Ni_3_S_2_. b) The local electron density distribution on the Pt site in the Pt_SA_–Ni_3_S_2_ system and the isosurface value is 0.0014 e A^−3^. c) Two‐dimensional (2D) isosurface map of the cross‐section consisting of Pt, S, and S atoms in Pt_SA_–Ni_3_S_2_ with a unit of e A^−3^. d) Calculated PDOS of Pt_SA_–Ni_3_S_2_, Pt_SA_–Ni_3_S_2_, and Pt foil with aligned Fermi level *E*_F_. e) The *d*‐*p* orbital alignment of the surface Pt, Ni sites for Pt_SA_–Ni_3_S_2_. f) Calculated *d*‐band center (*E*_d_) of Ni_3_S_2_, Pt_SA_‐Ni_3_S_2_, V_Ni_–Ni_3_S_2_, and Pt foil. g) Bader charge numbers of Pt atom in Pt_SA_–Ni_3_S_2_ and Pt foil. h) The free energy diagrams of H_2_O and H adsorbing on the surface of Pt_SA_–Ni_3_S_2_, Ni_3_S_2,_ and Pt foil.

First, theoretical investigations were carried out to elucidate the hidden mechanisms of Ni_3_S_2_ tailored Pt single atom on the electronic structure and HER process of catalyst. According to the optimized crystal structures (Figure S1, Supporting Information), the formation energy of Pt immobilized at surface Ni positions in Ni_3_S_2_ is −0.33 eV, significantly lower than that at S positions (7.83 eV), bottom Ni site (−0.23 eV), and surface S–S bridge site (−0.26 eV) in Ni_3_S_2_, suggesting the preference of surface cation occupancy of incorporated Pt. From the charge density difference analysis, the replacement of Ni site by Pt atom causes the redistribution of the local electrons around the Pt site by metal‐support coordination due to the different electronegativity among Pt (2.2), S (2.5), and S (2.5) (**Figure** **1**a,b; and Figures S2 and S3, Supporting Information), leading to a locally aggregated electrons area on the top of Pt site (Figure 1c), which is more favorable for boosting HER kinetics.^[^ ^19^ ^]^ The electrons redistribution could affect the partial density of states (PDOS) of the SACs. Compared with the Ni_3_S_2_ system, the total DOS （）of single‐atom Pt immobilized Ni_3_S_2_ show a higher electronic occupied state near the Fermi level (Figure 1d; and Figure S4, Supporting Information), leading to the higher intrinsic electrical conductivity and larger carrier concentration. Specifically, the improved DOS of the Pt_SA_‐Ni_3_S_2_ near the Fermi level mainly derives from the contribution of the Pt 5*d* orbitals electrons (Figure S5, Supporting Information), suggesting the strong orbital hybridization between S‐3*p* and Pt‐5*d* after introducing Pt into Ni position in the Ni_3_S_2_ system. Besides, the Pt‐5*d* band in Ni_3_S_2_ shows a substantially wide range for overlapping with H‐1*s* and H_2_O‐2*pπ* orbitals (Figure 1e). Meanwhile, the surface Ni‐3*d* band also exhibits optimal overlapping with H‐1*s* and H_2_O‐2*pπ* orbitals. Therefore, the anchored single‐atom Pt site plays a protecting role for stabilizing the Ni valence state against corrosion, and also acts as a distributary role for reducing the deactivation of reaction sites by binding H and O species under over‐binding of intermediates on the Ni‐sites.^[^ ^30^ ^]^ Further, the reactants‐support interaction in Pt_SA_‐Ni_3_S_2_ system was explored by performing the *d*‐band center model, and the Pt_SA_‐Ni_3_S_2_ shows the lowest *d*‐band center (−1.40 eV, Figure 1f)) comparing with that of Ni_3_S_2_ (−1.22 eV) and V_Ni_‐Ni_3_S_2_ (−1.26 eV), which could efficiently reduce the adsorption energy and meanwhile promote the desorption ability for H atoms on the catalyst surface.^[^ ^31^, ^32^ ^]^ Besides, the metal‐support interactions could be quantified by performing Bader charge analysis of the Pt atom (Figure 1g), and the charge transfer (absolute value of Bader charge value) between Pt atom and Ni_3_S_2_ support is only 0.017 e, much smaller than 0.044 e for Pt foil and ever lower than that of reported SACs recently (Table S2 (Supporting Information), 0.188 e for Pt_SA_ in CoSe_2−_ *_x_*,^[^ ^33^ ^]^ 0.23 e for Pt_SA_ in C_1_N_1_,^[^ ^13^ ^]^ and 0.47 e for Pt_SA_ in Co(OH)_2_),^[^ ^20^ ^]^ which suggests a metallic electron state for Pt atom anchoring in Ni_3_S_2_ support, leading to an extremely active catalytic property for HER.^[^ ^13^, ^18^ ^]^ From all of the above, the increased electrons occupation near Femi level, locally enhanced electrons density distribution on the top of Pt sites and shift‐down of the *d*‐band center deriving from the metallic‐state Pt induced by the orbital hybridization between S‐3*p* and Pt‐5*d* after introducing Pt into Ni position in Ni_3_S_2_ system are expected to optimize the reactant binding energy and promote the reaction kinetics for HER.

Thus, based on the optimized electronic structure of Pt_SA_‐Ni_3_S_2_ as the above analysis, the potential HER activity is predicted. Generally, the HER pathway consists of three main steps comprising the initial water adsorption H_2_O*, the generated intermediate H* as well as final molecular H_2_ (Figure 1h). The free energy (∆*G*_H*_) of hydrogen atom adsorption is a reasonable descriptor to estimate HER activity for various catalysts. According to the density functional theory (DFT) calculation, the ∆*G*_H*_ of Pt_SA_‐Ni_3_S_2_ (−0.06 eV) are closer to 0 eV than that of Ni_3_S_2_ (0.49 eV), Pt foil (−0.10 eV), and recently reported SACs (Table S2, Supporting Information), benefitting from the promoted electrons occupation near Femi level, down‐shift *d*‐band center as well as local‐enhance electrons density on Pt site due to the orbital hybridization between S‐3*p* and Pt‐5*d* after introducing Pt into Ni position in Ni_3_S_2_ system as above discussion. In the alkaline condition, the water molecular splitting kinetics from the Volmer step (step 2 in Figure 1h) determine the overall HER rate. Based on the DFT simulation, Pt_SA_‐Ni_3_S_2_ shows more favorable H_2_O adsorption with −0.67 eV comparing with Ni_3_S_2_, Pt foil, and recently reported SACs (Table S2, Supporting Information), and the improved H_2_O adsorption on Pt_SA_‐Ni_3_S_2_ site derives from the more *d* electrons as the real active centers of catalyst contributed by metallic‐state Pt 5*d* orbital (Figure 1g; and Figure S5, Supporting Information).^[^ ^13^ ^]^ Thus, transition‐metal based sulfide‐Ni_3_S_2_ immobilized Pt single atom provides an efficient approach to modulate the electronic structure of SACs and is predicted to show the most outstanding HER catalytic activity.

**Figure 2:**
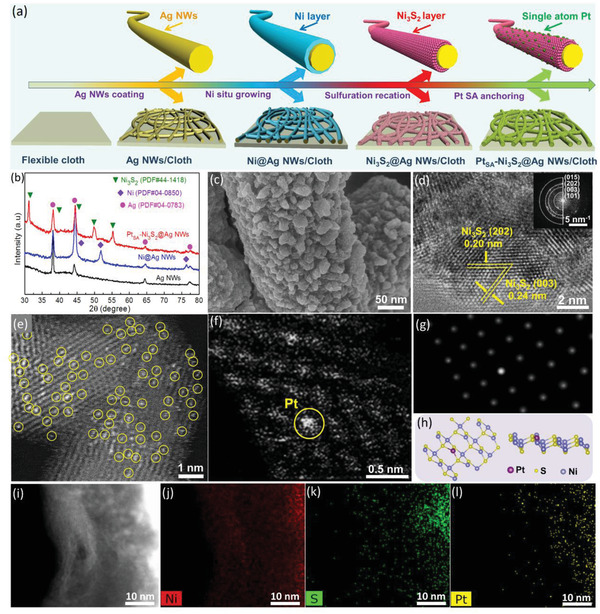
Fabrication and structural characterizations of Pt_SA_–Ni_3_S_2_@Ag NWs. a) Schematic illustration of the in‐situ preparation of Ni_3_S_2_ phase via sulfuration reaction of metallic Ni on Ag nanowires substrate followed by the electrochemical anchoring of single‐atom Pt. b) XRD patterns of bare Ag NWs, Ni@Ag NWs, and Pt_SA_–Ni_3_S_2_@Ag NWs. c) SEM images of Pt_SA_–Ni_3_S_2_@Ag NWs. d) HRTEM image of Pt_SA_–Ni_3_S_2_ and the corresponding FFT pattern of Pt_SA_–Ni_3_S_2_@Ag NWs in insert of c). e,f) Atomic‐resolution HAADF‐STEM images of Pt_SA_–Ni_3_S_2_. g) The simulated structure corresponding to the STEM image of Pt_SA_–Ni_3_S_2_ in e). h) The illustration of the DFT‐optimized structure of Pt_SA_–Ni_3_S_2_@Ag NWs in f). i–l) EDS element mapping of Pt_SA_–Ni_3_S_2_@Ag NWs.

Experimentally, we designed Ni_3_S_2_ immobilized Pt single‐atom catalyst with a 3D cable‐shape structure on cloth fiber‐loaded Ag NWs support (Pt_SA_‐Ni_3_S_2_@Ag NWs) by a four‐step route illustrated in **Figure** **2**a, in which Pt atoms immobilized at Ni positions in Ni_3_S_2_ is realized by Ni vacancies creation and subsequent Pt single atom anchoring. In detail, metal Ni was electrodeposited on Ag NWs by chronoamperometry method (Figure S6, Supporting Information). Then, the Ag NW supported Ni was converted into Ni_3_S_2_ (Ni_3_S_2_@Ag NWs) by the selective sulfuration process in Thioacetamide (TAA) aqueous solution. Finally, the formation of Ni vacancies and single‐atom Pt immobilization was performed by an electrochemical process with 400 cyclic voltammetry between 0 and −0.4 V in 1 m KOH solution with 50 × 10^−6^ m PtCl_6_^2−^.

The X‐ray diffraction (XRD) patterns (Figure 2b) confirm the phase evolution of metallic Ni to Ni_3_S_2_, and the diffraction peaks of Pt_SA_‐Ni_3_S_2_@Ag NWs mainly derive from Ni_3_S_2_ and metallic Ag. The absence of Pt characteristic peaks implies the single‐atom distribution of Pt instead of nanoparticles or clusters in Ni_3_S_2_. Scanning electron microscopy (SEM, Figure 2c; and Figure S7, Supporting Information) suggests the densely packed Pt_SA_‐Ni_3_S_2_ nanoparticles uniformly grow on Ag NWs to form a seamless contact nanoarchitecture via sulfuration and subsequent electrochemical process, which shows a different appearance with bare Ag NWs (Figure S8, Supporting Information) and Ni coated Ag NWs (Figure S9, Supporting Information). Further, the Pt_SA_‐Ni_3_S_2_@Ag NWs were characterized by transmission electron microscopy (TEM), and a distinct cable‐shape configuration is presented (Figure S10a, Supporting Information). Besides, interconnected pores were observed in the Pt_SA_‐Ni_3_S_2_ shell layer (Figure S10b, Supporting Information), which is favorable for hydrogen generation and release. The high‐resolution TEM (HRTEM, Figure 2d) image of Pt_SA_‐Ni_3_S_2_@Ag NWs demonstrates well‐resolved lattice fringes with 0.20 and 0.24 nm, corresponding to the exposed (202) and (003) crystallographic plane of Ni_3_S_2_, respectively. The derived fast Fourier transform (FFT) image (the inset in Figure 2d) also shows the related crystallographic plane of (101), (003), (202), and (015) from the Ni_3_S_2_ phase, suggesting the surface coupling of Pt‐support does not change the crystal structure of Ni_3_S_2_.

The high‐angle annular dark‐field scanning transmission electron microscopy (HAADF‐STEM) image presents uniformly dispersed bright spots on the Ni_3_S_2_ substrate (Figure 2e), corresponding to the heavy constituent atoms anchoring in Ni_3_S_2_, which verifies the formation of single‐atom Pt species. Furthermore, the atomic‐resolution HAADF‐STEM (Figure 2f; and Figure S10c,d, Supporting Information) demonstrates that the Pt atoms are fixed in Ni_3_S_2_. Subsequently, a STEM simulation based on the DFT‐optimized structure was performed to verify the microstructure of single‐atom Pt immobilized Ni_3_S_2_ (Figure 2g,h). The simulated result suggests that the single Pt atoms were anchored in the sites of Ni vacancies by binding with the nearest S atoms in Ni_3_S_2_ support. The corresponding energy‐dispersive spectroscopy (EDS) mapping analysis corroborates well the uniform dispersion of Pt atoms throughout the Ni_3_S_2_ (Figure 2i–l; and Figure S11, Supporting Information). While increasing the electrodeposition cyclic voltammetry (CV) cycle to 500 will result in the formation of Pt nanoclusters during the Pt immobilization process (Pt_NC_‐Ni_3_S_2_@Ag NWs, Figures S12–S14, Supporting Information), suggesting the continuous deposition of Pt atoms during the repeated polarization scanning, and the optimal HER catalytic performance could be obtained.

**Figure 3:**
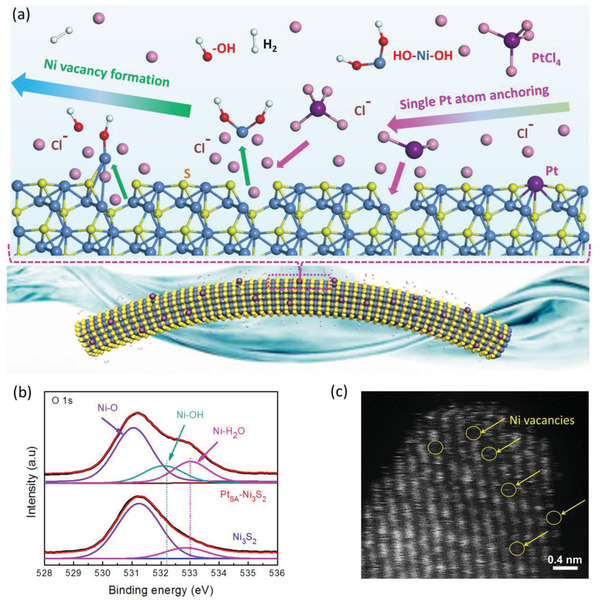
The design and mechanistic study for single‐atom Pt immobilized Ni_3_S_2_. a) The mechanism of the Ni vacancies formation and Pt single atom anchoring on Ni_3_S_2_. b) O 1*s* XPS spectra of Ni_3_S_2_ and Pt_SA_–Ni_3_S_2_. c) The HAADF‐STEM image of the V_Ni_–Ni_3_S_2_ obtained under the same conditions as Pt_SA_–Ni_3_S_2_ but replacing PtCl_6_^2−^ with NaCl in 1 m KOH solution.

The corresponding mechanism was unraveled by theoretical investigations and experimental characterization. As shown in **Figure** **3**a, the formation of Ni vacancies and Pt single atoms anchoring results from the facilitation of electrochemical reaction in alkaline media altering the chemical composition of Ni_3_S_2_@Ag NWs surface. Based on the theoretical calculation, the Ni‐terminated Ni_3_S_2_ provides the optimal site for water absorption (Figure 3b; and Figure S15, Supporting Information) and hydrogen evolution (Figure S16, Supporting Information). By capturing electrons, the Ni‐terminated basal planes are capable to reduce water into H_2_, and the generated −OH was grafted to the Ni terminal by the Ni—O band, causing the formation of Ni–OH intermediate confirmed by X‐ray photoelectron spectroscopy (XPS) (Figure 3b).^[^ ^34^, ^35^ ^]^ Further, the following reactions on Ni—OH intermediate include two possible routes. One is the restoration of Ni—OH intermediate to the original Ni—Ni_3_S_2_ terminal by the deabsorption of the −OH due to the weak binding between Ni and −OH comparing with the strong Ni—S bond.^[^ ^36^ ^]^ Another route involves the local corrosion of low‐valence Ni in Ni—OH intermediate supported by Ni_3_S_2_ induced by the chloride ions originating from PtCl_6_^2−^ in alkaline media to form high‐valence Ni^2+^—OH due to the strong depassivation ability and penetrating performance of Cl^−^ ions.^[^ ^37^, ^38^, ^39^ ^]^ Subsequently, by combining OH^−^ ions, the Ni^2+^—OH intermediate was transformed to the metal hydroxide Ni(OH)_2_ in the alkaline electrolyte to form a chemistry‐stable OH—Ni—OH leaving group.^[^ ^40^ ^]^ Consequently, the Ni vacancies on the surface of the Ni_3_S_2_ (V_Ni_–Ni_3_S_2_) were created for subsequent immobilization of Pt atoms. X‐ray near‐edge absorption spectra (XANES) for Ni K edge of V_Ni_–Ni_3_S_2_ are depicted in Figure S17a (Supporting Information) and show that the white line intensity of V_Ni_–Ni_3_S_2_ is lower than that of NiO, but higher than that of the pure Ni_3_S_2_. This proves that Ni atoms in V_Ni_–Ni_3_S_2_ are positively charged,^[^ ^7^, ^8^ ^]^ implying Ni vacancies formation. By Bader charge analysis deriving from DFT calculation (Figure S17b, Supporting Information), the average charge value of Ni atoms in V_Ni_–Ni_3_S_2_ (+0.28 e) is high than that of Ni atoms in Ni_3_S_2_ (+0.10 e), further implying the increased degree of nickel oxidization state and Ni vacancies formation. As shown in the HAADF‐STEM (Figure 3c; and Figure S17c, Supporting Information) image, after the same electrochemical process with Pt_SA_–Ni_3_S_2_@Ag NWs but replacing PtCl_6_^2−^ by NaCl in KOH solution to avoid the deposition of Pt species, the obtained Ni_3_S_2_ shows a large number of Ni vacancies (V_Ni_–Ni_3_S_2_). By comparing the STEM results of Pt_SA_–Ni_3_S_2_@Ag NWs and V_Ni_–Ni_3_S_2_@Ag NWs, the Pt atoms are finely anchored in the sites of Ni vacancies. Combining the analysis in theoretical investigations (Figure 1e), the anchored Pt atoms at Ni positions under cathodic potential will protect adjacent Ni against corrosion. Consequently, a stable single‐atom Pt‐Ni_3_S_2_ support system could be obtained. Besides, the release of reduced H_2_ will serve as the dynamic template to generate interconnected pores in the Ni_3_S_2_ layer,^[^ ^25^ ^]^ leading to more accessible basal planes for Ni vacancies defects creation and Pt atoms immobilization.

**Figure 4:**
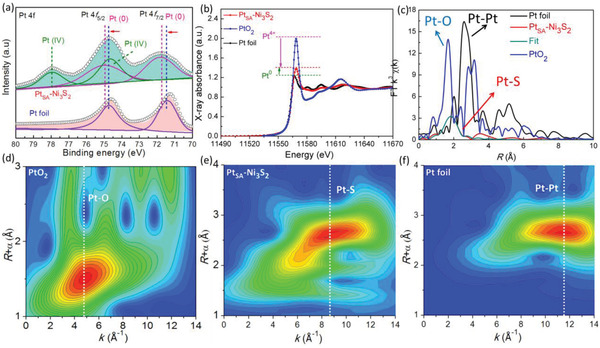
Electronic state identification of single‐atom Pt in Ni_3_S_2_. a) Pt 4*f* XPS spectra of Pt_SA_–Ni_3_S_2_ and Pt foil. b) Experimental Pt *K*‐edge XANES and c) FT‐EXAFS of Pt *K*‐edge EXAFS signal (without phase correction). d–f) WT‐EXAFS of Pt_SA_–Ni_3_S_2_ and reference samples.

Further, the electronic states of Pt atoms in Pt_SA_–Ni_3_S_2_ were verified by XPS, X‐ray absorption near‐edge spectroscopy (XANES), and extended X‐ray absorption fine spectroscopy (EXAFS) measurements. The binding energy in high‐resolution Pt 4*f* XPS spectra (**Figure** **4**a) shows a slightly positive shift for Pt_SA_–Ni_3_S_2_ compared to Pt foil. Moreover, the Ni 2*p* and S 2*p* XPS peaks show an inapparent evolution in the binding energy after Pt anchoring (Figure S18, Supporting Information), suggesting a weak electrons transfer between Pt atoms and Ni_3_S_2_ support. The corresponding electronic states of Pt atoms in Pt_SA_–Ni_3_S_2_ were further explored by XANES measurements (Figure 4b), in which Pt_SA_–Ni_3_S_2_ show slightly enhanced intensity than Pt foil but extremely low than PtO_2_, proving less electron loss of Pt atoms in Ni_3_S_2_ as theoretical calculation in Figure 1g.^[^ ^13^ ^]^ The Fourier transforms spectrum of the *k*3‐weighted EXAFS oscillations of Pt_SA_–Ni_3_S_2_ exhibits a prominent peak in the region 1.8–2.4 Å (Figure 4c), corresponding to the Pt–Cl and Pt–S first coordination shell.^[^ ^41^, ^42^ ^]^ The absence of Pt–Pt contribution peaks at about 2.7 Å confirms atomically dispersed Pt in Ni_3_S_2_. The fitting results of the Fourier transform curves give a coordination number of 3 for Pt–S contribution (Figure 4c; and Table S1, Supporting Information), confirming that the single Pt atoms were anchored in the sites of Ni vacancies by binding with the nearest three S atoms in Ni_3_S_2_ support (Figure S1c, Supporting Information). To further verify the coordination conditions of Pt atoms, the wavelet transforms (WT, Figure 4d–f) analysis was performed, which can provide powerful resolution in the radial distance in the *R* and *K* space. A WT intensity maximum at about 5 and 12 Å^−1^ are attributed to the Pt–O and Pt–Pt contributions, respectively.^[^ ^43^ ^]^ In contrast, the intensity maximum of Pt_SA_–Ni_3_S_2_ is near 8 Å^−1^, assigned to the Pt–S contribution, and the Pt–Pt coordination with an intensity maximum at ≈12 Å^−1^ is not detected, confirming the presence of isolated Pt atoms in Ni_3_S_2_.

**Figure 5:**
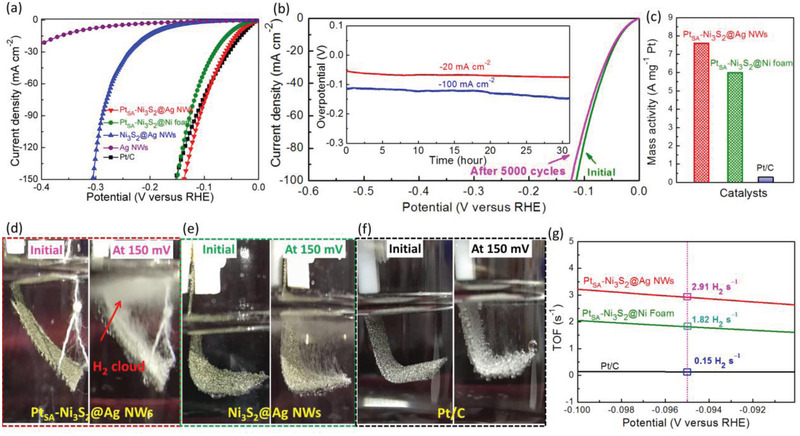
Electrochemical performance measurement. a) Linear sweep voltammograms (LSV) polarization curves of Pt_SA_–Ni_3_S_2_@Ag NWs, Pt_SA_–Ni_3_S_2_@Ni foam, Ni_3_S_2_@Ag NWs, Ag NWs, and Pt/C@Ni foam. b) Stability test of Pt_SA_–Ni_3_S_2_@Ag NWs via multicycle potential scanning and chronoamperometry method (Inset in Figure 5b). c) The mass activity of Pt atom in Pt_SA_–Ni_3_S_2_@Ag NWs, Pt_SA_–Ni_3_S_2_@Ni foam, and state‐of‐the‐art Pt/C on Ni foam. The digital photos of fabricated catalysts d) Pt_SA_–Ni_3_S_2_@Ag NWs, e) Pt_SA_–Ni_3_S_2_@Ni foam, and f) Pt/C@Ni foam at the initial state and an overpotential of 150 mV in 1 m KOH electrolyte for HER. g) TOF plots of the Pt_SA_–Ni_3_S_2_@Ag NWs, Pt_SA_–Ni_3_S_2_@Ni foam, and Pt/C@Ni foam after normalizing to Pt atom sites.

Based on the theoretical calculation and structural characterizations, for the first time, we reveal that Ni_3_S_2_ tuned single‐atom Pt tends to present a promising activity for HER by the integration of electronic structure adjustment strategy and architectural nanostructure engineering. Experimentally, the electrocatalytic performance of seamlessly conductive Pt_SA_–Ni_3_S_2_@Ag NWs with 3D architecture for HER was verified in alkaline electrolyte, and the original Ni_3_S_2_@Ag NWs, Ag NWs, and Ni foam supported commercial Pt/C (Pt/C@Ni foam) were also measured for comparison. Besides, Pt_SA_–Ni_3_S_2_@Ni foam was fabricated under the same conditions as Pt_SA_–Ni_3_S_2_@Ag NWs but replacing Ag NWs with Ni Foam. As demonstrated in **Figure** **5**a, the Pt_SA_–Ni_3_S_2_@Ag NWs shows the highest HER performance among all catalysts, and only needs 33 and 137 mV to achieve the current density of 10 and 150 mA cm^−2^, respectively, suggesting significantly superior activity than the Pt_SA_–Ni_3_S_2_@Ni foam, Ni_3_S_2_@Ag NWs, Ag NWs, and even the commercial Pt/C on Ni foam at an overpotential of >80 mV (Figure S19, Supporting Information), in which the decrease of the current density of Pt/C at the high potential should be attributed to the application of Nafion as the binder and poor nanostructure morphology of Pt/C electrode, hindering the rapid generation and release of H_2_. Besides, the Pt_SA_–Ni_3_S_2_@Ag NWs exhibit a similar Tafel slope (34.70 mV dec^−1^) with Pt_SA_–Ni_3_S_2_@Ni foam and Pt/C@Ni foam towards HER (Figure S20, Supporting Information), suggesting Pt‐like kinetics deriving from the introduction of single‐atom Pt. Moreover, the single‐atom Pt anchored Ni_3_S_2_@Ag NWs also possess higher activity than the Ni_3_S_2_@Ag NWs supported Pt particles (Figure S12, Supporting Information) due to the more efficient synergy of the single Pt atom and the Ni_3_S_2_ host. Apart from the outstanding metal‐support interaction, the Ag NWs are introduced to provide facile electron transport pathways through the entire catalyst, and consequently, the charge transfer resistance (*R*_ct_) between Pt_SA_–Ni_3_S_2_@Ag NWs and reactants (H_2_O*, M–H_abs_ and H*) reach a significantly low value of 0.95 Ω (Figure S21, Supporting Information), leading to a faster faradaic reaction.^[^ ^26^, ^27^ ^]^ Moreover, Pt_SA_–Ni_3_S_2_@Ag NWs exhibits a 5.1‐time increase in double‐layer capacitance (*C*_dl_) over Ni_3_S_2_@Ag NWs (Figure S22, Supporting Information), suggesting more exposed sites for Pt atoms immobilization and HER due to the formation of a large number of voids in Ni_3_S_2_ during Pt anchoring process (Figure S10, Supporting Information). Considering the actual applications, Pt_SA_–Ni_3_S_2_@Ag NWs also shows excellent stability with negligible decay in HER activity for 5000 cycles or 30 h (Figure 5b). The corresponding structural measurements including SEM, STEM images, EDS mapping, XRD, and XPS data (Figures S24 and S25, Supporting Information) are performed to confirm the single‐atom dispersion and intact structure of Pt_SA_–Ni_3_S_2_@Ag NWs after the stability test, suggesting the high stability of Ni_3_S_2_@Ag NWs immobilized single‐atom Pt by Ni vacancies. Furthermore, the mass activity of Pt_SA_–Ni_3_S_2_@Ag NWs normalized to the Pt loading mass (1.47 wt%, measured by inductively coupled plasma‐mass spectrometry) at an overpotential of 150 mV is 7.6 A mg^−1^ (Figure 5c), which is 27‐fold high than that of the commercial Pt/C catalyst (20 wt% Pt/C, 0.28 A mg^−1^). As a result, a large hydrogen generation rate was observed at room temperature for Pt_SA_–Ni_3_S_2_@Ag NWs (Figure 5d) comparing with Pt_SA_–Ni_3_S_2_@Ni foam (Figure 5e) and Pt/C@Ni foam (Figure 5f). Especially, an aggregative H_2_ cloud was presented on the top of the Pt_SA_–Ni_3_S_2_@Ag NWs electrode, indicating the high efficiency of M–H_ads_ generation and facile conversion to H_2_. Besides, comparing with Ni foam supported commercial Pt/C electrode (Figure 5f) fabricated by using Nafion as the binder, no large bubble absorbs on the surface of Pt_SA_–Ni_3_S_2_@Ag NWs electrode, which could be attributed to the shift‐down of the *d*‐band center (Figure 1f), more optimal ∆G_H*_ with −0.06 eV than that of Pt foil (−0.10 eV, Figure 1h), as well as the formation of abundant voids (Figure 2c; and Figures S10 and S23, Supporting Information) of Ni_3_S_2_ immobilized single‐atom Pt coating on Ag NWs network for facile desorption and release of H_2_. To quantify the output efficacy of H_2_, the turnover frequency (TOF, Note S1, Supporting Information) per Pt atom was performed. As shown in Figure 5g, Pt_SA_–Ni_3_S_2_@Ag NWs catalyst shows a high TOF value of 2.91 H_2_ s^−1^, which is 1.6 and 19.4 times higher than that of Pt_SA_–Ni_3_S_2_@Ni foam and Pt/C@Ni foam, respectively, demonstrating the extremely high activity of Pt_SA_–Ni_3_S_2_@Ag NWs for HER. For the Faradaic efficiency, we roughly collect the H_2_ by a drainage method to estimate the experimental gas amount. Faradaic efficiency can be determined by the ratio of H_2_ (experimental) to H_2_ (theoretical) in a percentage form. To determine the Faradaic efficiency of the Pt_SA_–Ni_3_S_2_@Ag NWs hybrid catalyst for HER, the electrolysis was performed in 1 m KOH with an applied constant current density of 100 mA cm^−2^ for 100 s. The Faradaic efficiency of about 90% was obtained for HER, indicating that the generated charges were almost consumed for H_2_ generation. As summarized in Table S2 (Supporting Information), our Pt_SA_–Ni_3_S_2_@Ag NWs also show superior HER performances than the recently reported SACs, confirming that the improved HER activity of the fabricated Pt_SA_–Ni_3_S_2_@Ag NWs mainly derives from the more favorable reconstruction of the electronic‐state structure after single‐atom Pt anchoring on Ni_3_S_2_ support.

## 3 Conclusion

Based on the theoretical simulation, we design a transition‐metal sulfide‐Ni_3_S_2_, with abundant exposed Ni vacancies created by the interaction between chloride ions and the functional groups on the surface of Ni_3_S_2_, to anchor atomically dispersed Pt for efficiently electrocatalytic hydrogen evolution. Specifically, Ni_3_S_2_ tailored Pt single atom shows a weak metal‐support interaction, increasing the *d* orbital electron occupation near Femi level and shift‐down of the *d*‐band center for optimizing the reactants absorption during HER. The unique coordination environment of Pt sites is constructed by the orbital hybridization between S‐3*p* and Pt‐5*d* after introducing Pt into Ni position in Ni_3_S_2_ system, inducing a locally enhanced electronic field distribution by increasing the electron density and metallic‐state feature on the Pt sites, promoting the electrochemical HER kinetics. By integrating single‐atom modification strategy and architectural nanostructure engineering, the fabricated 3D Pt_SA_–Ni_3_S_2_@Ag NWs exhibits outstanding HER catalytic activity in terms of high turnover frequencies (2.91 H_2_ s^−1^ at 95 mV) and extremely great mass activity of 7.6 A mg^−1^, exceeding the commercial Pt/C catalyst and the advanced SACs reported recently. This work presents a new avenue for developing high‐activity single‐atom electrocatalyst by tailoring the electronic structure and geometrical construction of SACs via transition‐metal based sulfides.

## 4 Experimental Section

##### Synthesis of Ni@Ag NWs

First, an oil bath process was carried out to synthesize Ag nanowires (Ag NWs). In detail, an ethylene glycol solution containing FeCl_3_ (7.19 × 10^−3^ m), AgNO_3_ (0.051 m), and polyvinylpyrrolidone (0.012 m) was kept at 110 °C for 12 h by oil bath pan. Then, the generated yellow precipitate was collected and washed with acetone and alcohol to obtain Ag NWs. Subsequently, the cleaned Ag NWs were dispersed and stored in an alcohol solution. A conductive network was fabricated by spray coating Ag NWs on a flexible cloth fabric. Finally, the metallic Ni layer is grown on Ag NWs network by a facile electrodeposition process. Specifically, the solution consisting of 0.10 m NiCl_2_, 0.09 m H_3_BO_3,_ and a solvent containing ethanol and deionized water with 2:5 in volume ratio. Then the electrodeposition of metallic Ni was performed by chronoamperometry with 1.2 V for 400 s in a standard three‐electrode system on a workstation (CHI660E), in which graphite sheet acted as a counter electrode, saturated calomel electrode (SCE) acted as a reference electrode. Finally, the Ag NWs supported metallic Ni was cleaned and dried for stand‐by.

##### Synthesis of Ni_3_S_2_@Ag NWs

Ni_3_S_2_@Ag NWs were prepared by performing a hydrothermal treatment on obtained Ni@Ag NWs. In brief, the Ni@Ag NWs coated cloth pieces were immersed in a 50 mL Teflon‐reaction vessel containing 30 mL of 3 mmol TAA aqueous solution and then kept at 120 °C for 90 min. The obtained samples were cleaned with water and alcohol, and finally drying at room temperature, marking as Ni_3_S_2_@Ag NWs material.

##### Synthesis of V_Ni_–Ni_3_S_2_@Ag NWs

V_Ni_–Ni_3_S_2_@Ag NWs were fabricated by an electrochemical process in the three‐electrode system, in which Ni_3_S_2_@Ag NWs coated cloth was performed as the working electrode, graphite sheet acted as a counter electrode, SCE acted as a reference electrode. The corresponding electrochemical process was carried out by multicycle cathode polarization in 1 m KOH solution containing 0.5 m NaCl with a scan rate of 50 mV s^−1^ between 0.00 and −0.50 V versus reversible hydrogen electrode (RHE) for 400 cycles.

##### Synthesis of Pt_SA_–Ni_3_S_2_@Ag NWs

Pt_SA_–Ni_3_S_2_@Ag NWs were fabricated by an electrochemical process in the three‐electrode system, in which Ni_3_S_2_@Ag NWs coated cloth was performed as the working electrode, graphite sheet acted as a counter electrode, SCE acted as a reference electrode. The corresponding electrochemical process was carried out by multicycle cathode polarization in 1 m KOH solution containing 50 × 10^−6^ m H_2_PtCl_6_ with a scan rate of 50 mV s^−1^ between 0.00 and −0.50 V versus RHE for 400 cycles.

##### Characterizations

The morphology of the fabricated catalysts in this study was observed by SEM (GeminiSEM 300). HRTEM images, HAADF‐STEM images, and STEM‐EDX mapping images were obtained by a TEM coupled with an energy spectrum analyzer (JEOL JEM2100). The Pt contents in Pt_SA_–Ni_3_S_2_@Ag NWs were measured by inductively coupled plasma optical emission spectrometry. The XPS spectra of elements in Pt_SA_–Ni_3_S_2_@Ag NWs were tested by a surface analysis system (ESCALAB250Xi). The phase and crystal information of the fabricated catalysts were obtained by Cu *Kα* radiation in an XRD (Shimadzu). The EXAFS measurement of the Pt_SA_–Ni_3_S_2_@Ag NWs at the Pt *L*_3_‐edge was performed at 1W1B station at the Beijing Synchrotron Radiation Facility. The cloth‐supported Pt_SA_–Ni_3_S_2_@Ag NWs were directly subjected to measurements. Data analysis and fitting were performed with Athena and Artemis in the Demeter package.

##### Electrochemical Measurements

All electrochemical tests were finished by an electrochemical workstation (CHI 660E) with a three‐electrode configuration, in which fabricated catalysts in this work were employed as the working electrode, graphite sheet acted as a counter electrode, SCE acted as a reference electrode. All the presented potential in this work was calibrated versus RHE according to the experimental method.^[^ ^13^ ^]^ HER performance tests were performed in 1 m KOH aqueous solution. Linear sweep voltammograms (LSV) with 95% iR‐corrections were tested with a potential range from 0.05 to −0.5 V under a scan rate of 5 mV s^−1^. Electrochemical impedance spectroscopy (EIS) was obtained by a frequency range from 100 k to 0.1 Hz with 200 mV of initial voltage. For the preparation of Pt/C@Ni foam, 5 mg 20 wt% Pt/C was dispersed in 0.9 mL alcohol containing 0.1 mL 5 wt% Nafion solution to form a homogeneous ink. Then, the obtained ink was coated on the Ni foam and dried in air to form a porous Pt/C@Ni foam electrode.

##### DFT Theoretical Calculations

All the DFT simulations were performed by the Vienna Ab‐initio Simulation Package (VASP)^[^ ^44^, ^45^, ^46^ ^]^ with the projector‐augmented‐wave method.^[^ ^47^, ^48^ ^]^ And the method DFT‐D3 developed by Grimme et al.^[^ ^49^, ^50^ ^]^ was also employed to account for the dispersion correction and van der Waals' interaction into the conventional Kohn–Sham DFT potential energy calculation. The exchange‐correlation energy was described by the Perdew–Burke–Ernzerhof (PBE) implementation of generalized gradient approximation (GGA‐PBE).^[^ ^51^ ^]^ Spin‐polarized calculations have been performed with plane wave expansion truncated at 500 eV. 2 × 4 × 1 *k*‐mesh grids were set for the self‐consistent field calculation and structural relaxation. Electronic relaxation utilized the conjugate‐gradient (CG)^[^ ^52^ ^]^ with the energy convergence being 10^−5^ eV. Geometry optimization was conducted by using the quasi‐Newton algorithm^[^ ^53^, ^54^ ^]^ with residual forces on atoms low than 0.01 eV Å^−1^. In all calculations, spin polarization was considered. A vacuum region thickness of 20 Å was built. The atoms at the top two layers were free for structural relaxation.

The H and H_2_O absorbing on Ni_3_S_2_, Pt_SA_–Ni_3_S_2,_ and Pt slab were investigated by comparing the formation energy of different sites. The equation for calculating adsorption enthalpy Δ*E*_H*_ as the following

(1) $$\Delta E_{H^{*}}=E_{\mathrm{slab}+H}-E_{\mathrm{slab}}-\frac{1}{2}E_{H_{2}}$$

Where the *E_slab+H_* is the total enthalpy of H adsorbing on the catalysts, the enthalpy of the catalysts is *E_slab_*, the H_2_ enthalpy is *E*_H2_. As similar, the equation for calculating the H_2_O adsorption enthalpy ∆*E*_H2O_ as the following

(2) $$\Delta E_{H_{2}O^{*}}=-E_{\mathrm{slab}+H_{2}O}-E_{\mathrm{slab}}-E_{H_{2}O^{*}}$$

The free energy of adsorbed H and H_2_O as follows

(3) $$\Delta G_{H^{*}}=\Delta E_{H^{*}}+\Delta E_{\mathrm{ZPE}}-T\Delta S$$

(4) $$G_{H_{2}O^{**}}=\Delta E_{H_{2}O^{*}}+\Delta E_{\mathrm{ZPE}}-T\Delta S$$

where Δ*E*_H*_ represent the H adsorption energy and ∆*E*_H2O*_ represent the H_2_O adsorption energy, and Δ*E*_ZPE_ represents the difference related to the zero‐point energy between the gas phase and the adsorbed state.

## Conflict of Interest

The authors declare no conflict of interest.

## Supporting information

Supporting Information

## Data Availability

Research data are not shared.

## References

[1] M. K. Debe , Nature 2012, 486, 43. 22678278 10.1038/nature11115

[2] J. Staszak‐Jirkovský , C. D. Malliakas , Nat. Mater. 2016, 15, 197. 26618882 10.1038/nmat4481

[3] M. A. Legare , G. Belanger‐Chabot , R. D. Dewhurst , E. Welz , I. Krummenacher , B. Engels , H. Braunschweig , Science 2018, 359, 896. 29472479 10.1126/science.aaq1684

[4] B. Yu , H. Li , J. White , S. Donne , J. Yi , S. Xi , Y. Fu , H. Yu , Z. Chen , T. Ma , Adv. Funct. Mater. 2020, 30, 1905665.

[5] N. Cheng , S. Stambula , D. Wang , M. N. Banis , J. Liu , A. Riese , B. Xiao , R. Li , T. K. Sham , L. M. Liu , Nat. Commun. 2016, 7, 13638. 27901129 10.1038/ncomms13638 PMC5141386

[6] L. Zhang , R. Si , H. Liu , N. Chen , Q. Wang , K. Adair , Z. Wang , J. Chen , Z. Song , J. Li , Nat. Commun. 2019, 10, 4936. 31666505 10.1038/s41467-019-12887-y PMC6821730

[7] P. Li , M. Wang , X. Duan , L. Zheng , X. Cheng , Y. Zhang , Nat. Commun. 2019, 10, 1711. 30979899 10.1038/s41467-019-09666-0 PMC6461613

[8] W. H. Lai , L. F. Zhang , W. B. Hua , S. Indris , Z. C. Yan , Z. Hu , B. Zhang , Y. Liu , L. Wang , M. Liu , R. Liu , Y. X. Wang , J. Z. Wang , Z. Hu , H. K. Liu , S. L. Chou , S. X. Dou , Angew. Chem., Int. Ed. 2019, 58, 11868. 10.1002/anie.201904614 31173428

[9] C. H. Choi , M. Kim , H. C. Kwon , S. J. Cho , S. Yun , H. T. Kim , K. J. J. Mayrhofer , H. Kim , M. Choi , Nat. Commun. 2016, 7, 10922. 26952517 10.1038/ncomms10922 PMC4786782

[10] J. Liu , M. Jiao , B. Mei , Y. Tong , Y. Li , M. Ruan , P. Song , G. Sun , L. Jiang , Y. Wang , Z. Jiang , L. Gu , Z. Zhou , W. Xu , Angew. Chem., Int. Ed. 2018, 58, 1163. 10.1002/anie.201812423 30520205

[11] L. Cao , Q. Luo , W. Liu , Y. Lin , X. Liu , Y. Cao , W. Zhang , Y. Wu , J. Yang , T. Yao , Nat. Catal. 2019, 2, 134.

[12] L. Xuning , L. Liu , X. Ren , G. Jiajian , Y. Huang , B. Liu , Sci. Adv. 2020, 6, eabb6833.32967833

[13] S. Fang , X. Zhu , X. Liu , J. Gu , W. Liu , D. Wang , W. Zhang , Y. Lin , J. Lu , S. Wei , Nat. Commun. 2020, 11, 1029. 32098951 10.1038/s41467-020-14848-2 PMC7042219

[14] J. Zhang , Y. Zhao , X. Guo , C. Chen , C. L. Dong , R. S. Liu , C. P. Han , Y. Li , Y. Gogotsi , G. Wang , Nat. Catal. 2018, 1, 985.

[15] J. Wan , W. Chen , C. Jia , L. Zheng , J. Dong , X. Zheng , Y. Wang , W. Yan , C. Chen , Q. Peng , D. Wang , Y. Li , Adv. Mater. 2018, 30, 1705369. 10.1002/adma.201705369 29363197

[16] H. Li , L. Wang , Y. Dai , Z. Pu , Z. Lao , Y. Chen , M. Wang , X. Zheng , J. Zhu , W. Zhang , Nat. Nanotech. 2018, 13, 411. 10.1038/s41565-018-0089-z 29556007

[17] N. J. O'Connor , A. Jonayat , M. J. Janik , T. P. Senftle , Nat. Catal. 2018, 1, 531.

[18] M. T. Greiner , T. Jones , S. Beeg , L. Zwiener , M. Scherzer , F. Girgsdies , S. Piccinin , M. Armbrüster , A. Knop‐Gericke , R. Schlögl , Nat. Chem. 2018, 10, 1008. 30150725 10.1038/s41557-018-0125-5

[19] D. Liu , X. Li , S. Chen , H. Yan , C. Wang , C. Wu , Y. Haleem , S. Duan , J. Lu , B. Ge , P. Ajayan , Y. Luo , J. Jiang , L. Song , Nat. Energy 2019, 4, 1.

[20] K. Zhou , C. Han , C. Wang , Z. Wang , Q. Zhang , X. Ke , J. Liu , H. Wang , Energy Environ. Sci. 2020, 13, 3082.

[21] S. Ye , F. Luo , Q. Zhang , P. Zhang , T. Xu , D. He , L. Guo , Y. Zhang , C. He , X. Ouyang , Energy Environ. Sci. 2019, 12, 1000.

[22] L. Zhuang , Y. Jia , H. Liu , X. Wang , X. Yao , Adv. Mater. 2018, 31, 1805581.

[23] Y. Y. Chen , Y. Zhang , X. Zhang , T. Tang , H. Luo , S. Niu , Z. H. Dai , L. J. Wan , J. S. Hu , Adv. Mater. 2017, 29, 1703311. 10.1002/adma.201703311 28833679

[24] S. H. Bae , J. E. Kim , H. Randriamahazaka , S. Y. Moon , J. Y. Park , I. K. Oh , Adv. Energy Mater. 2017, 7, 1601492.

[25] K. Zhou , Q. Zhang , Z. Wang , C. Wang , C. Han , X. Ke , Z. Zheng , H. Wang , J. Liu , H. Yan , J. Mater. Chem. A 2019, 7, 26566.

[26] J. X. Feng , J. Q. Wu , Y. X. Tong , G. R. Li , J. Am. Chem. Soc. 2017, 140, 610. 10.1021/jacs.7b08521 29058435

[27] J. Zhang , R. Yanzhang , M. Du , Q. Wang , G. Gao , J. Wu , G. Wu , M. Zhang , B. Liu , J. Yao , X. Zhang , Adv. Mater. 2015, 27, 4752. 26179503 10.1002/adma.201501969

[28] P. Zhang , L. Li , D. Nordlund , H. Chen , L. Fan , B. Zhang , X. Sheng , Q. Daniel , L. Sun , Nat. Commun. 2018, 9, 381. 29374160 10.1038/s41467-017-02429-9 PMC5786058

[29] K. Zhou , Q. Zhang , J. Liu , H. Wang , Y. Zhang , J. Mater. Chem. A 2020, 8, 4524.

[30] J. Yin , J. Jin , M. Lu , B. Huang , H. Zhang , Y. Peng , P. Xi , C. H. Yan , J. Am. Chem. Soc. 2020, 142, 18378. 32955265 10.1021/jacs.0c05050

[31] Z. Chen , Y. Song , J. Cai , X. Zheng , D. Han , Y. Wu , Y. Zang , N. Shuwen , Y. Liu , J. Zhu , X. Liu , Angew. Chem., Int. Ed. 2018, 57, 5076. 10.1002/anie.201801834 29498161

[32] S. Ye , F. Luo , Q. Zhang , P. Zhang , T. Xu , Q. Wang , D. He , L. Guo , Y. Zhang , C. He , Energy Environ. Sci. 2019, 12, 1000.

[33] L. Zhuang , Y. Jia , H. Liu , X. Wang , R. Hocking , H. Liu , J. Chen , L. Ge , L. Zhang , M. Li , C. L. Dong , Y. C. Huang , D. Yang , Z. Zhu , X. Yao , Adv. Mater. 2018, 31, 1805581. 10.1002/adma.201805581 30488551

[34] Z. Cai , L. Li , Y. Zhang , Z. Yang , J. Yang , Y. Guo , L. Guo , Angew. Chem., Int. Ed. 2019, 58, 4189. 10.1002/anie.201812601 30672090

[35] Q. Qian , Y. Li , Y. Liu , L. Yu , G. Zhang , Adv. Mater. 2019, 31, 1901139. 10.1002/adma.201901139 30972836

[36] B. Fei , Z. Chen , J. Liu , H. Xu , X. Yan , H. Qing , M. Chen , R. Wu , Adv. Energy Mater. 2020, 10, 2001963.

[37] S. Dong , E. L.a Plante , X. Chen , M. Torabzadegan , M. Balonis , M. Bauchy , G. Sant , Mater. Degradat. 2018, 2, 32.

[38] E. Doná , M. Cordin , C. Deisl , E. Bertel , C. Franchini , R. Zucca , J. Redinger , J. Am. Chem. Soc. 2009, 131, 2827. 19203185 10.1021/ja809674n

[39] S. Hao , L. Chen , C. Yu , B. Yang , Z. Li , Y. Hou , L. Lei , X. Zhang , ACS Energy Lett. 2019, 4, 952.

[40] M. Morcillo , B. Chico , J. Alcántara , I. Díaz , J. Simancas , D. Fuente , Mater. Corros. 2014, 66, 882. 10.3390/ma10040406 PMC5506973 28772766

[41] J. Deng , H. Li , J. Xiao , Y. Tu , D. Deng , H. Yang , H. Tian , J. Li , P. Ren , X. Bao , Energy Environ. Sci. 2015, 8, 1594.

[42] Y. Wang , Y. Feng , Y. Guan , Z. Jiang , X. Gu , H. Zhang , Z. Huang , J. Li , J. Mater. Chem. A 2018, 6, 11783.

[43] G. Cui , Nat. Commun. 2019, 10, 5812. 31862887 10.1038/s41467-019-13685-2 PMC6925196

[44] G. Kresse , Comput. Mater. Sci. 1996, 6, 15.

[45] G. Kresse , Phys. Rev. B 1996, 54, 11169. 10.1103/physrevb.54.11169 9984901

[46] G. Kresse , Phys. Rev. B 1994, 49, 14251. 10.1103/physrevb.49.14251 10010505

[47] P. Blöchl , Phys. Rev. B 1994, 50, 17953. 10.1103/physrevb.50.17953 9976227

[48] G. Kresse , Phys. Rev. B 1999, 59, 1758.

[49] J. Klimeš , D. R. Bowler , A. Michaelides , J. Phys.: Condens. Matter 2009, 22, 022201. 21386245 10.1088/0953-8984/22/2/022201

[50] J. Klimeš , D. R. Bowler , A. Michaelides , Phys. Rev. B 2011, 83, 195131.

[51] J. P. Perdew , K. Burke , Phys. Rev. Lett. 1996, 77, 3865. 10062328 10.1103/PhysRevLett.77.3865

[52] M. C. Payne , M. P. Teter , D. C. Allan , T. Arias , a. J. Joannopoulos , Rev. Modern Phys. 1992, 64, 1045.

[53] M. Methfessel , A. Paxton , Phys. Rev. B 1989, 40, 3616. 10.1103/physrevb.40.3616 9992329

[54] P. Pulay , Chem. Phys. Lett. 1980, 73, 393.

