# Supplementary material for: Atomically Dispersed Platinum Modulated by Sulfide as an Efficient Electrocatalyst for Hydrogen Evolution Reaction
Source: Adv Sci (Weinh). 2021 May 13;8(12):2100347. doi: 10.1002/advs.202100347 (PMC8224416; doi:10.1002/advs.202100347)
Supplement: Supplementary file 1 — Supporting Information [file ADVS-8-2100347-s001.pdf]

WILEY-VCH

© 2021 Wiley-VCH GmbH

ADVANCED SCIENCE
Open Access

Supporting Information

for *Adv. Sci.,* DOI: 10.1002/advs.202100347

Atomically Dispersed Platinum Modulated by Sulfide as an Efficient Electrocatalyst for Hydrogen Evolution Reaction

Kai Ling Zhou, Chang Bao Han, Zelin Wang, Xiaoxing Ke, Changhao Wang, Yuhong Jin, Qianqian Zhang, Jingbing Liu, Hao Wang* and Hui Yan*

WILEY-VCH

© 2021 Wiley-VCH GmbH

Supporting Information

Atomically Dispersed Platinum Modulated by Sulfide as an Efficient Electrocatalyst for Hydrogen Evolution Reaction

Kai Ling Zhou, Chang Bao Han, Zelin Wang, Xiaoxing Ke, Changhao Wang, Yuhong Jin, Qianqian Zhang, Jingbing Liu, Hao Wang* and Hui Yan*

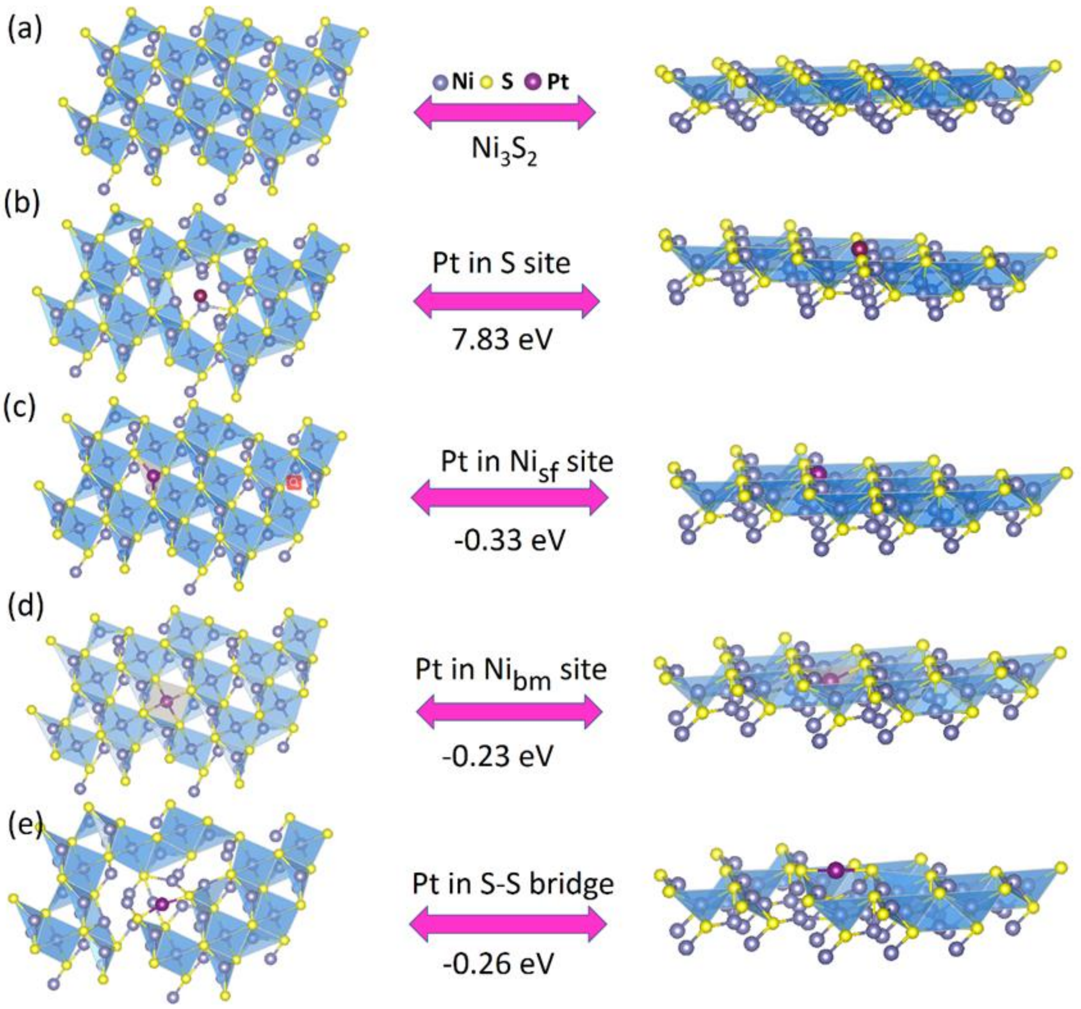

Figure S1. The optimized structures of (a) Ni_3S_2 and the formation energy of Pt immobilized at the (b) surface S site (c) surface Ni site, (d) bottom Ni site, and (e) surface S-S bridge site in Ni_3S_2.

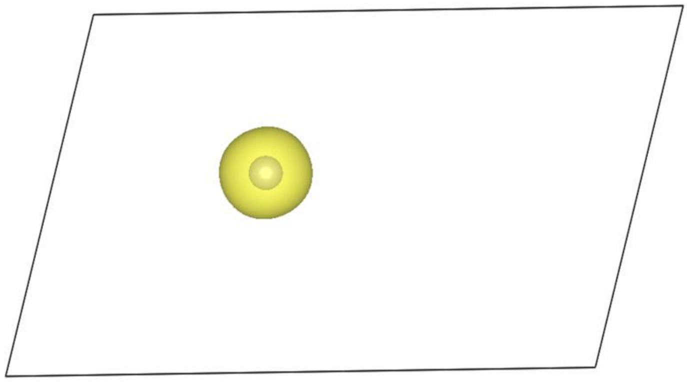

Figure S2. The charge density distribution on the isolated Pt atom without coordination with Ni_3S_2.

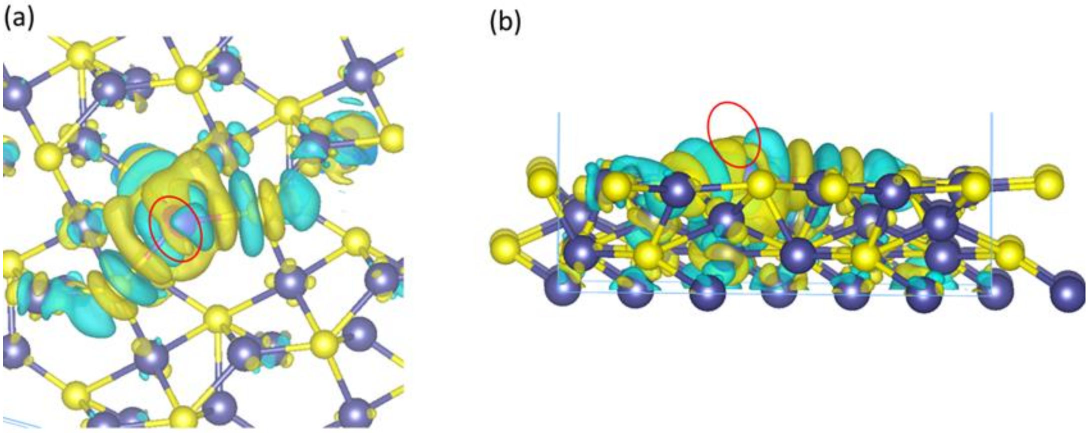

Figure S3. The magnified electron density distribution on the Pt site in the Pt_{SA}-Ni_3S_2 system with different views.

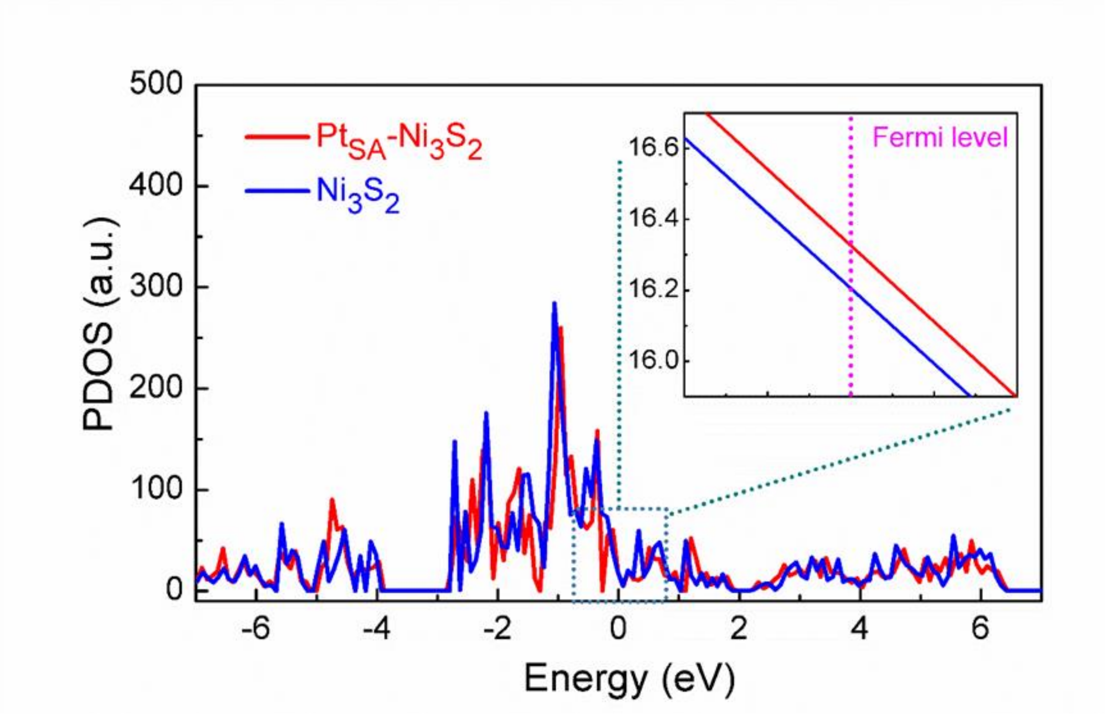

Figure S4. Highlighted total DOS of Ni_3S_2 and Pt_{SA}-Ni_3S_2 with aligned Fermi level.

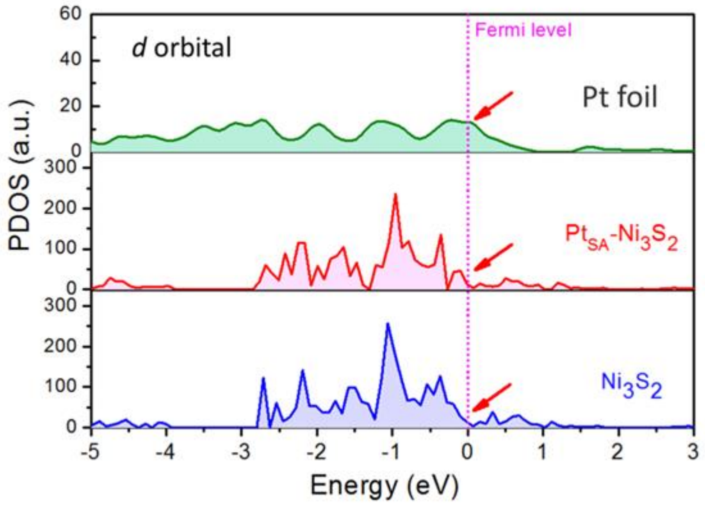

Figure S5. Calculated PDOS of *d* orbitals for Ni_3S_2, Pt_{SA}-Ni_3S_2, and Pt foil.

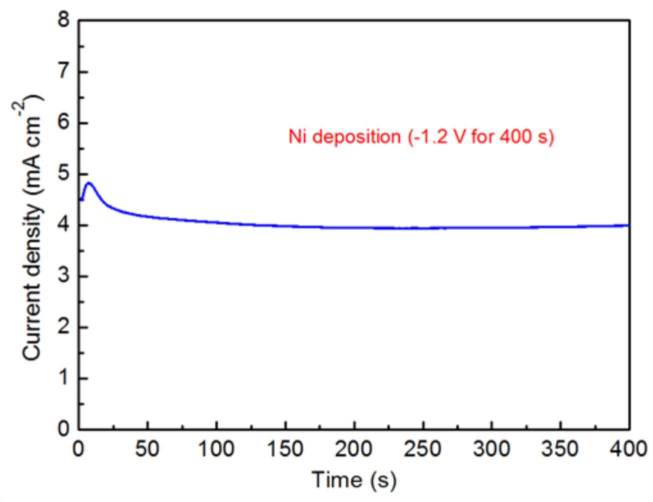

Figure S6. Chronoamperometry curve for metal Ni deposition on Ag NWs network.

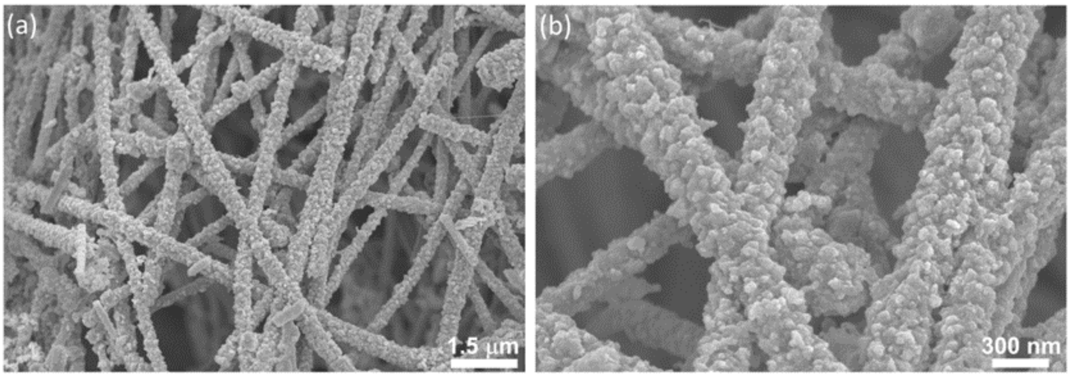

Figure S7. SEM images of Pt_{SA}-Ni_3S_2@Ag NWs with different magnifications.

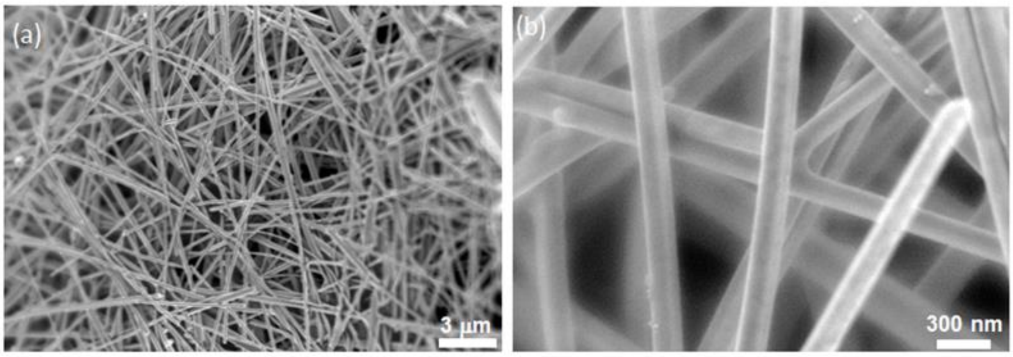

Figure S8. SEM images of Ag NWs with different magnifications.

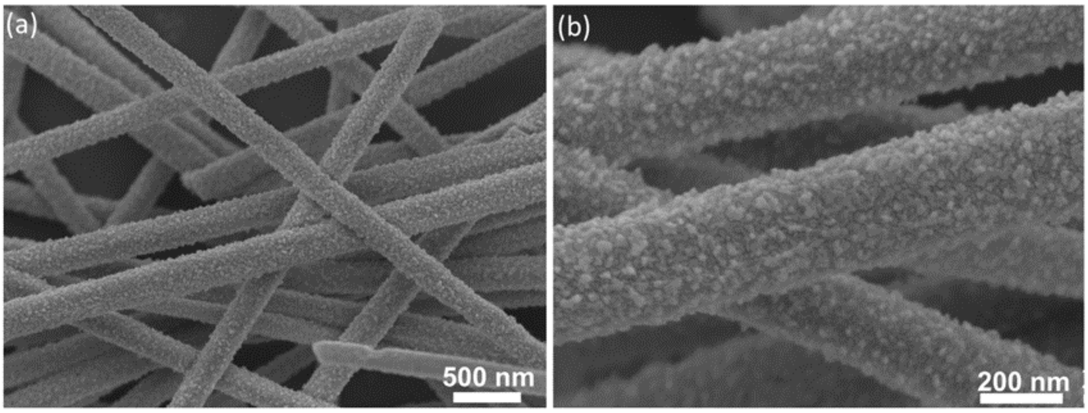

Figure S9. SEM images of Ni@Ag NWs with different magnifications.

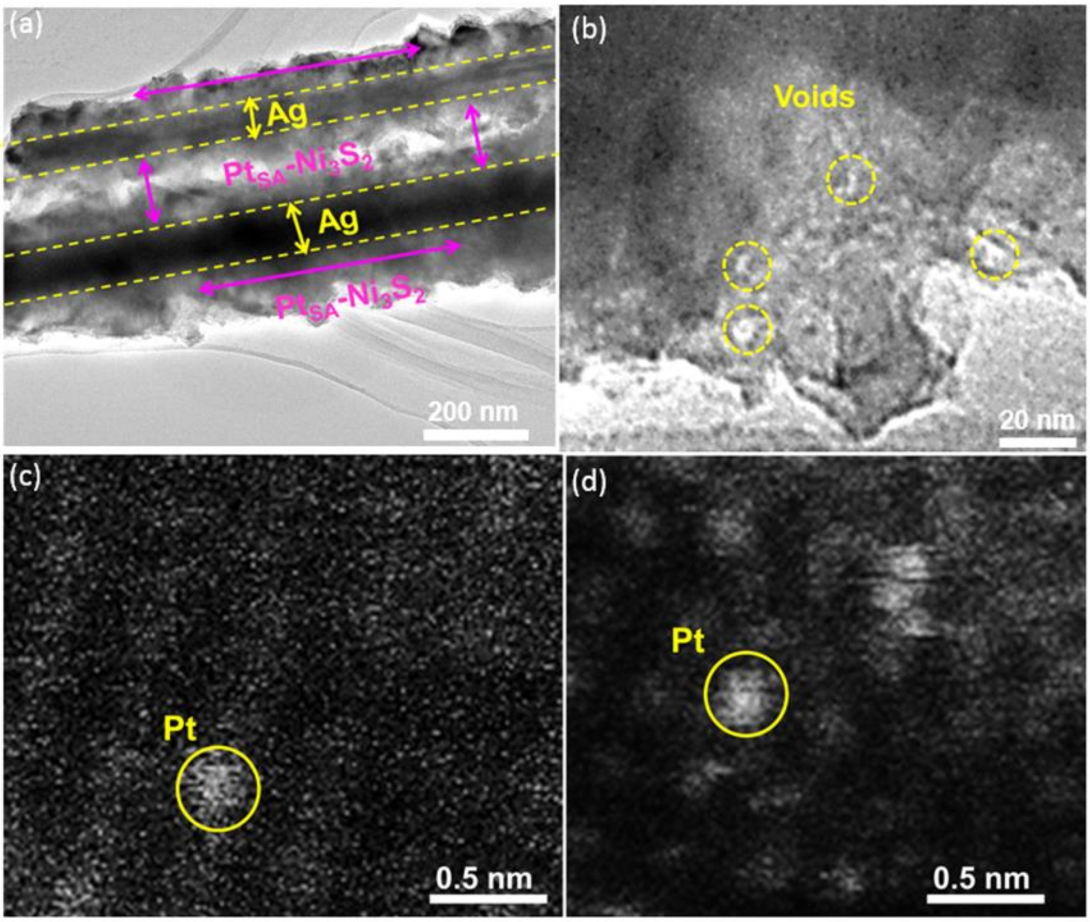

Figure S10. (a-b) TEM images of Pt_{SA}-Ni_3S_2@Ag NWs with different magnifications. (c-d) Atomic-resolution HAADF-STEM images of Pt_{SA}-Ni_3S_2 with different areas.

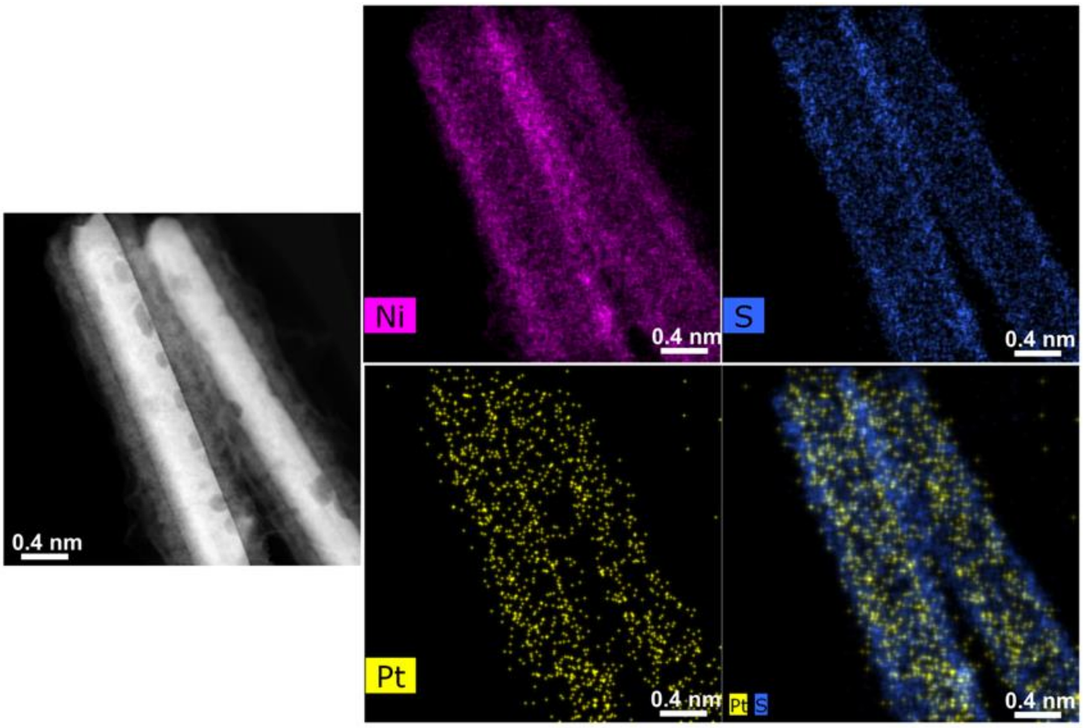

Figure S11. STEM element mapping of Pt_{SA}-Ni_3S_2@Ag NWs at low magnification.

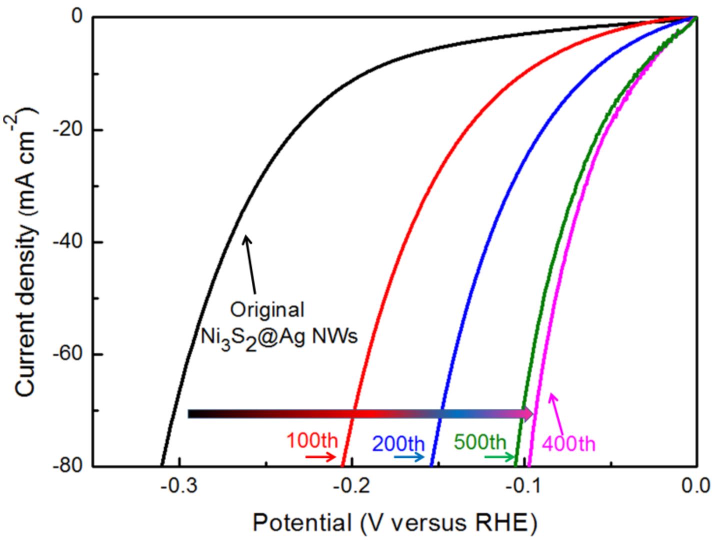

Figure S12. HER CV polarization curves of Ni_3S_2@Ag NWs at the different potential cycles for Pt atom immobilization.

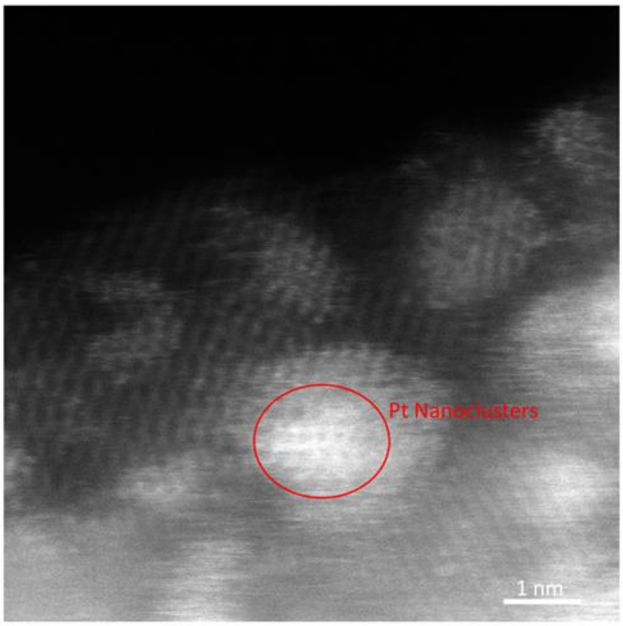

Figure S13. STEM image of Pt_{NC}-Ni_3S_2@Ag NWs after cycling for 500 cycles.

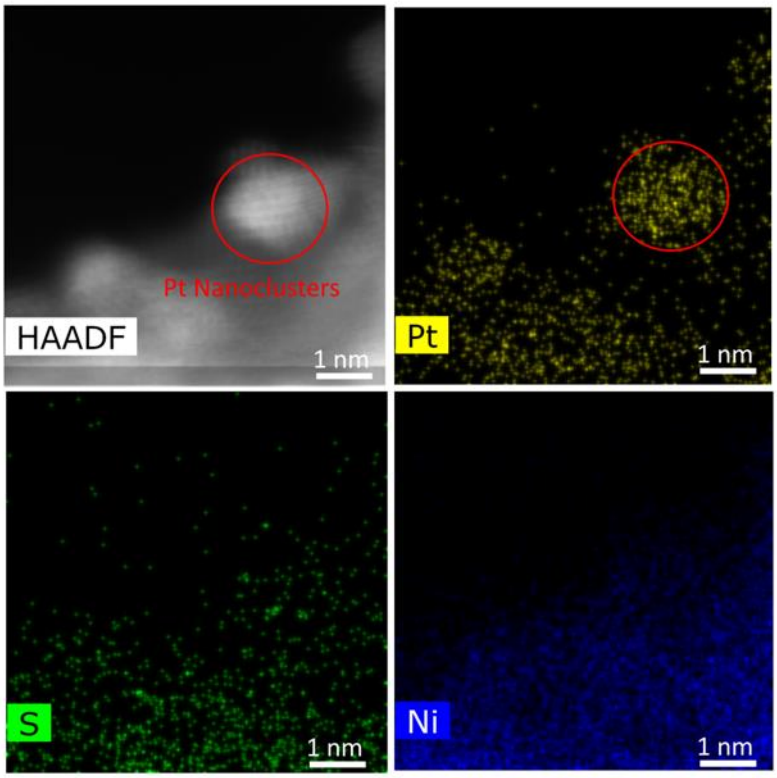

Figure S14. EDS mapping of Pt_{NC}-Ni_3S_2@Ag NWs after cycling for 500 cycles.

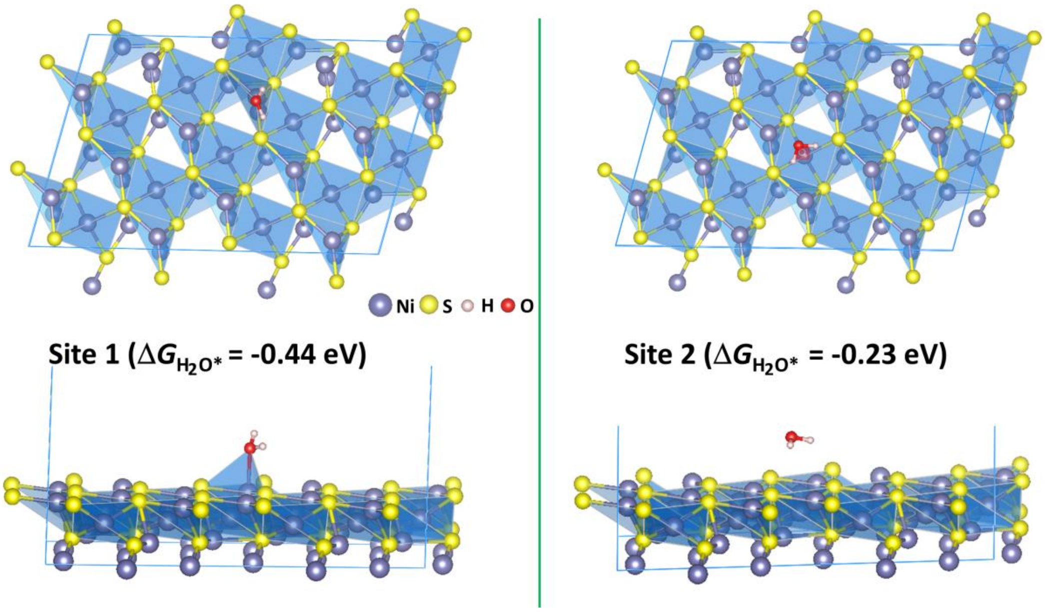

Figure S15. The H_2O adsorption energy of the different sites on Ni_3S_2.

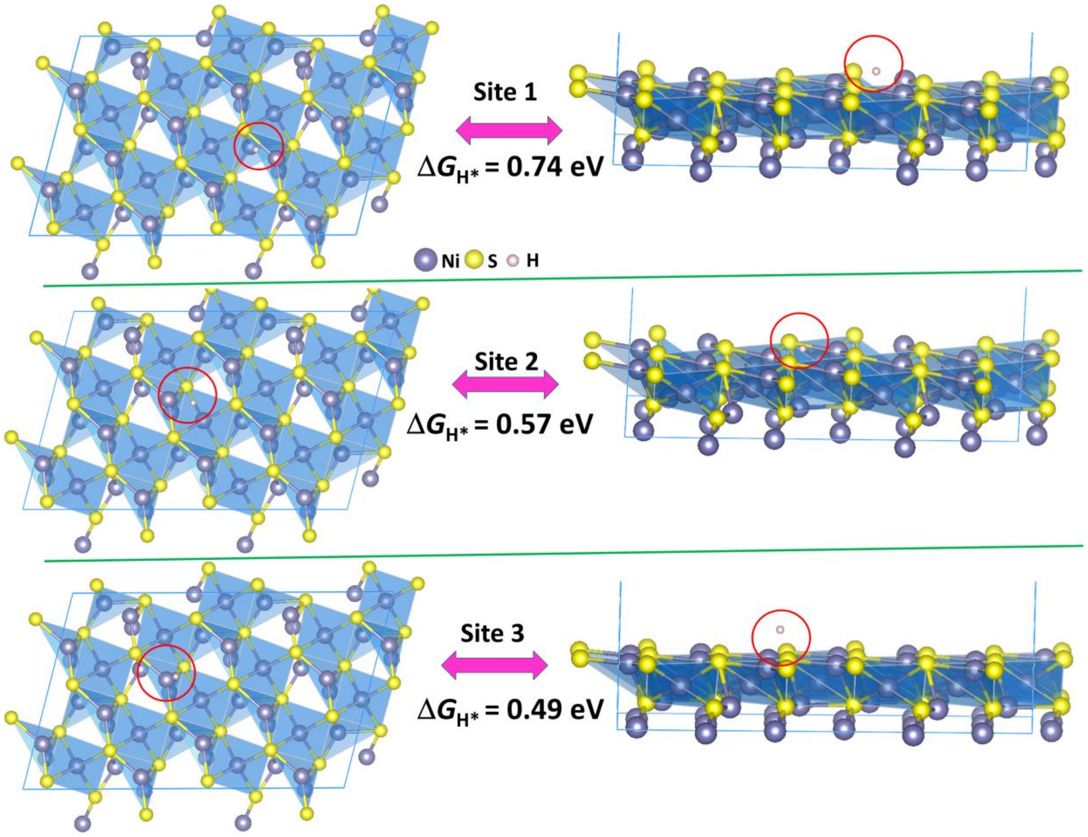

Figure S16. The free energy (ΔG_{H*}) for hydrogen adsorption on the different sites of Ni_3S_2.

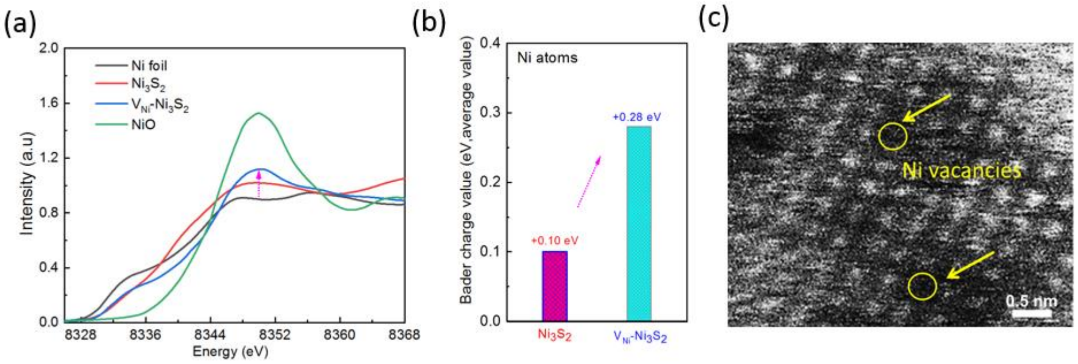

Figure S17. (a) The Ni *K* edge XANES spectra. (b) The average value of Bader charge numbers of Ni atoms in V_{Ni}-Ni_3S_2 and Ni_3S_2. (c) The HAADF-STEM image of the V_{Ni}-Ni_3S_2 obtained under the same conditions as Pt_{SA}-Ni_3S_2 but replacing $PtCl_6^{2-}$ with NaCl in 1 M KOH solution.

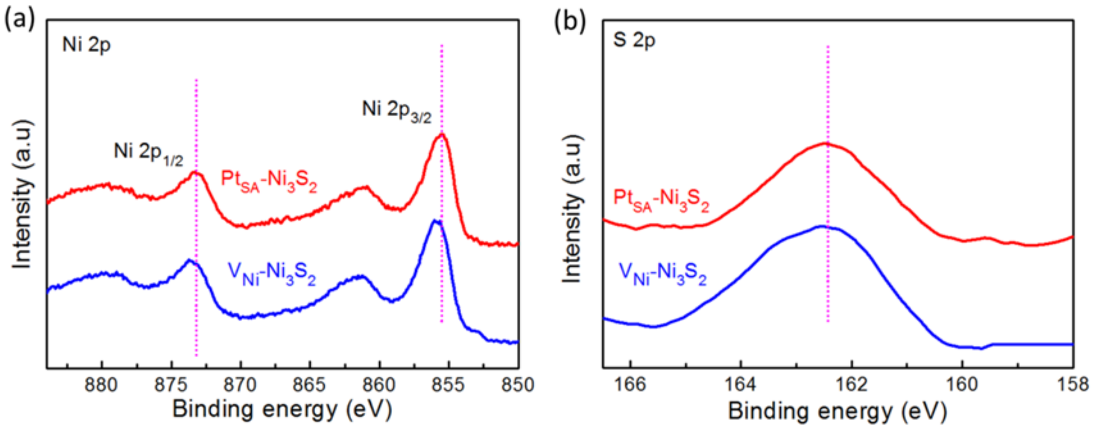

Figure S18. (a) Ni 2*p* and (b) S 2*p* XPS spectra of Pt_{SA}-Ni_3S_2 and V_{Ni}-Ni_3S_2.

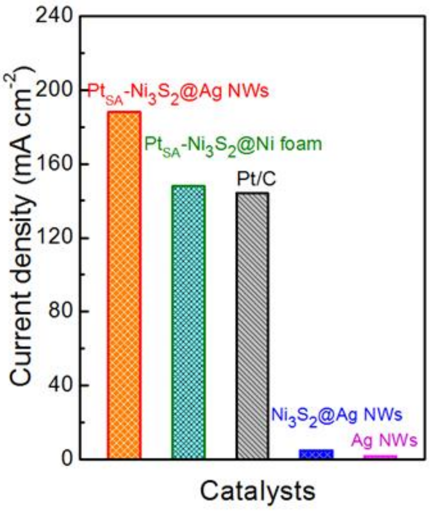

Figure S19. The comparison of the achieved current densities for various catalysts at an overpotential of 150 mV.

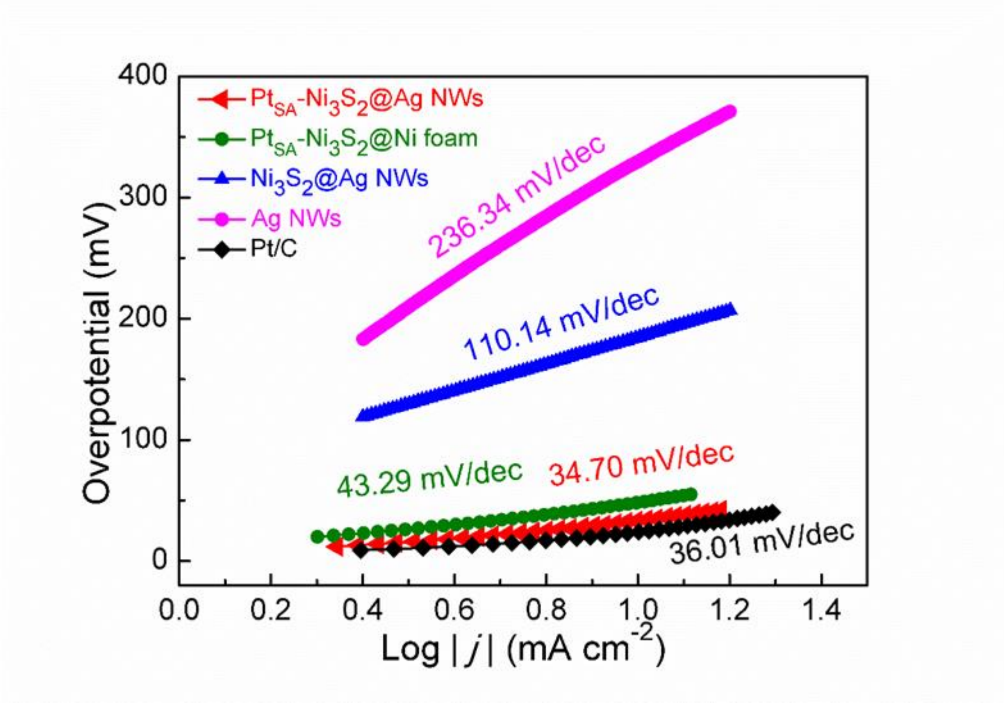

Figure S20. Tafel slope deriving from the related LSV curves in Figure 5a.

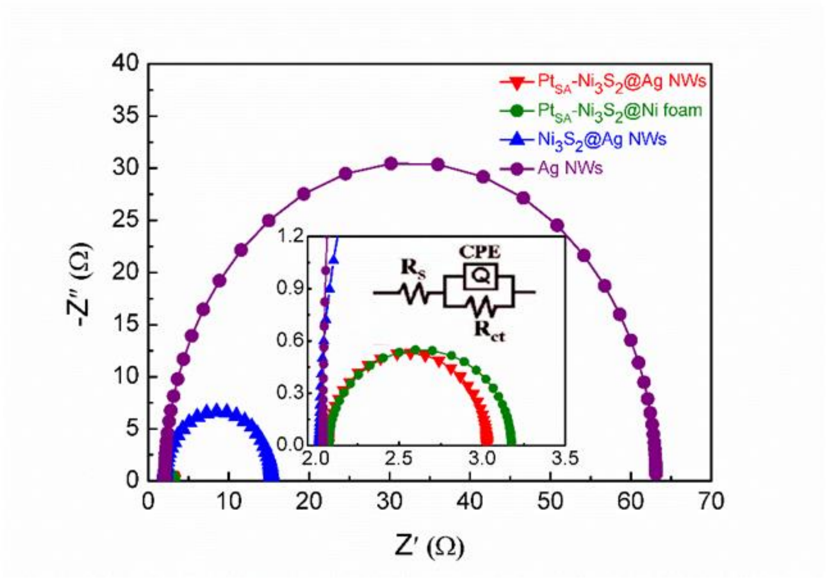

Figure S21. Electrochemical impedance spectroscopy (EIS) Nyquist plots of Pt$_{SA}$-Ni$_3$S$_2$@Ag NWs, Pt$_{SA}$-Ni$_3$S$_2$@Ni foam, Ni$_3$S$_2$@Ag NWs, and Ag NWs.

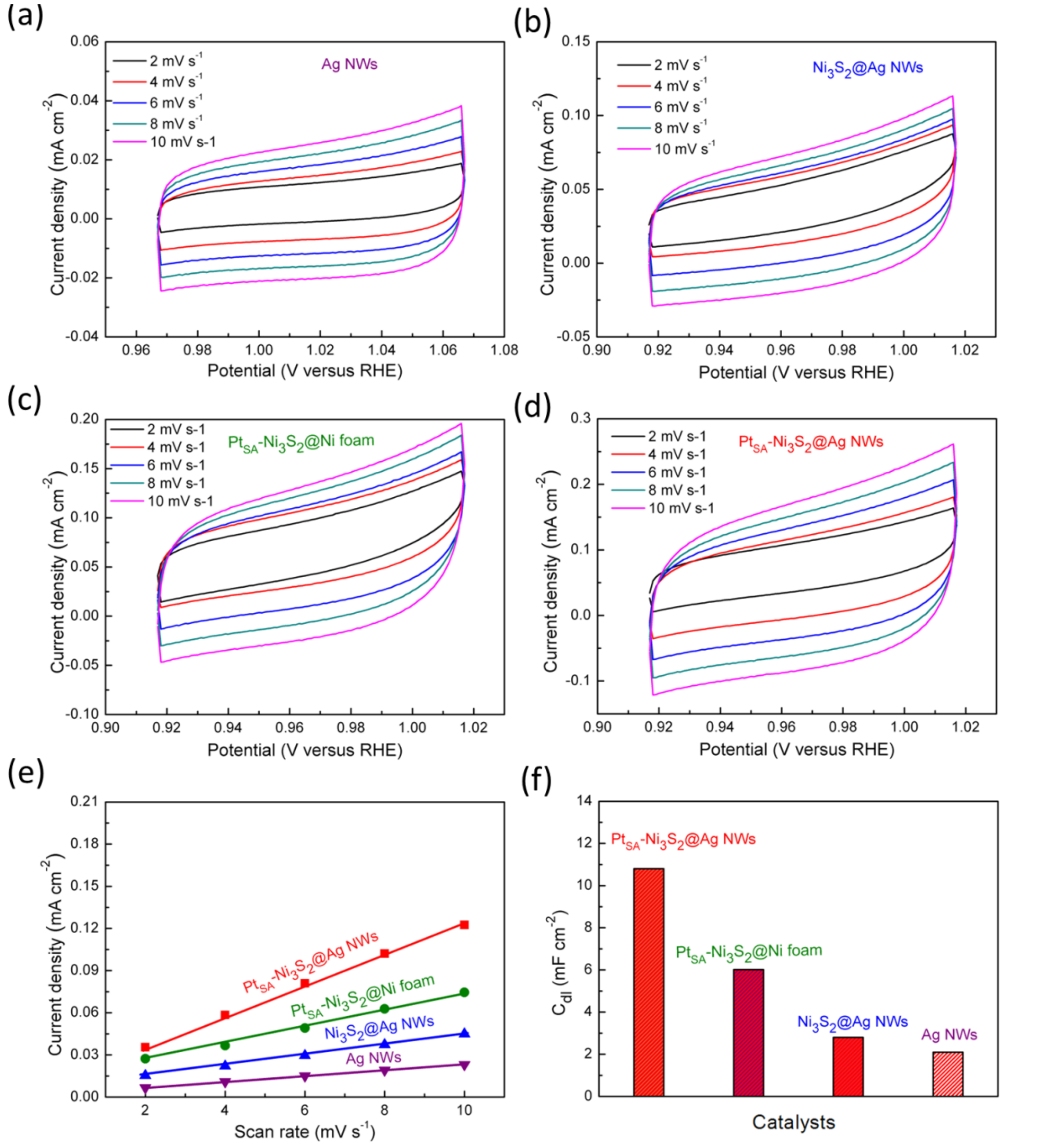

Figure S22. Cyclic voltammograms are carried out for (a) Ag NWs, (b) N_3S_2@Ag NWs, (c) Pt_{SA}-Ni_3S_2@Ni foam, and (d) Pt_{SA}-Ni_3S_2@Ag NWs in a potential range without faradaic reaction participation. (e). The corresponding average current as a function of the scan rates. (f) The comparison of C_{dl} between various catalysts.

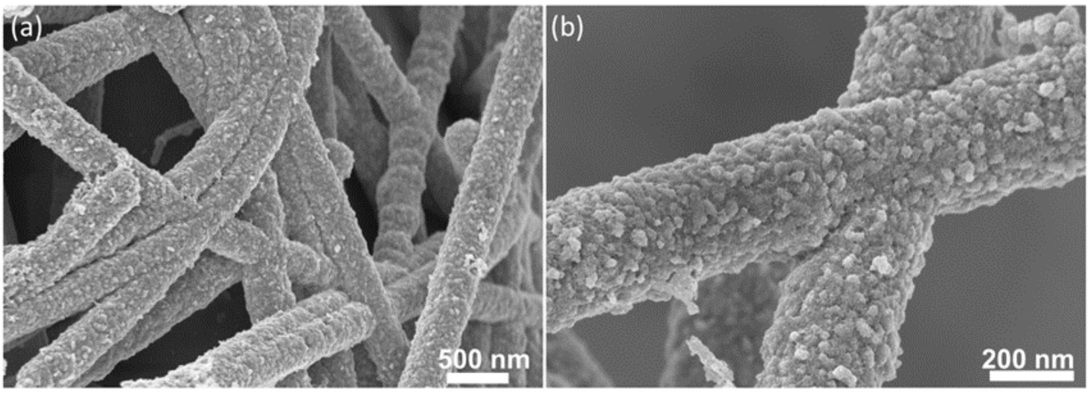

Figure S23. SEM images of Ni_3S_2@Ag NWs with different magnifications.

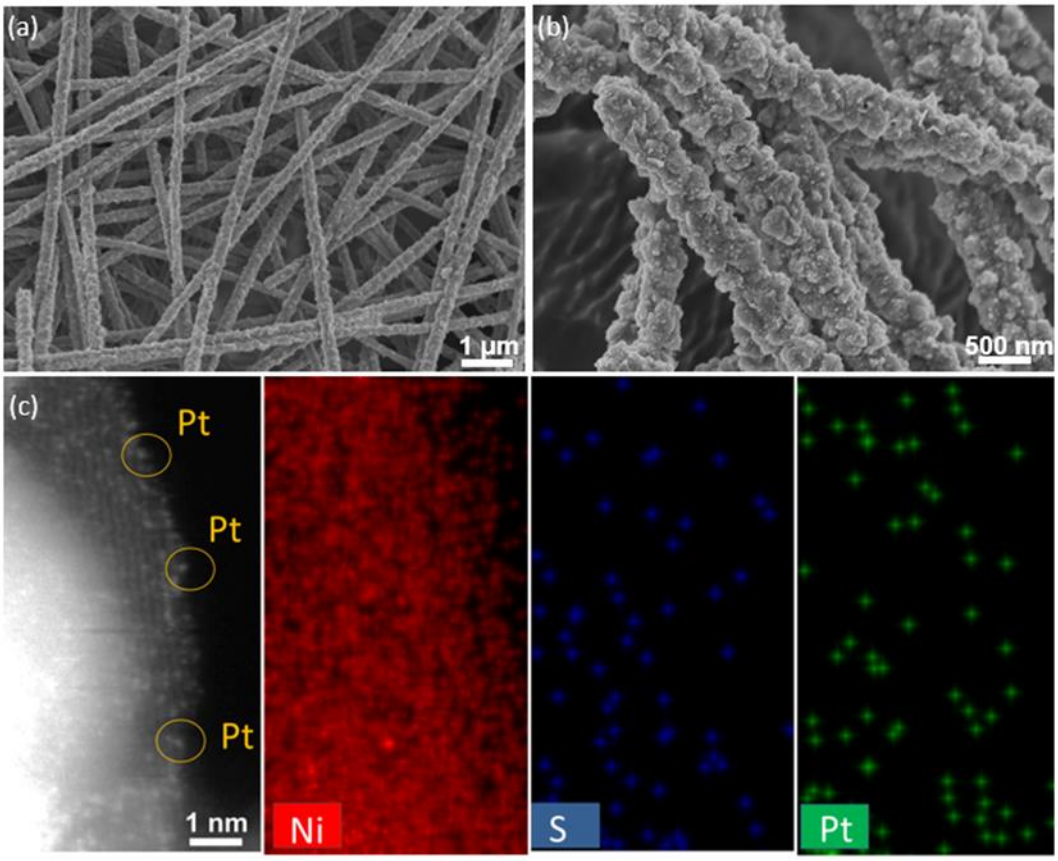

Figure S24. Characterization of the Pt_{SA}-Ni_3S_2@Ag NWs after stability test. (a-b) SEM images, and (c) atomic-resolution HAADF-STEM images and EDS mapping of Pt_{SA}-Ni_3S_2@Ag NWs after stability test.

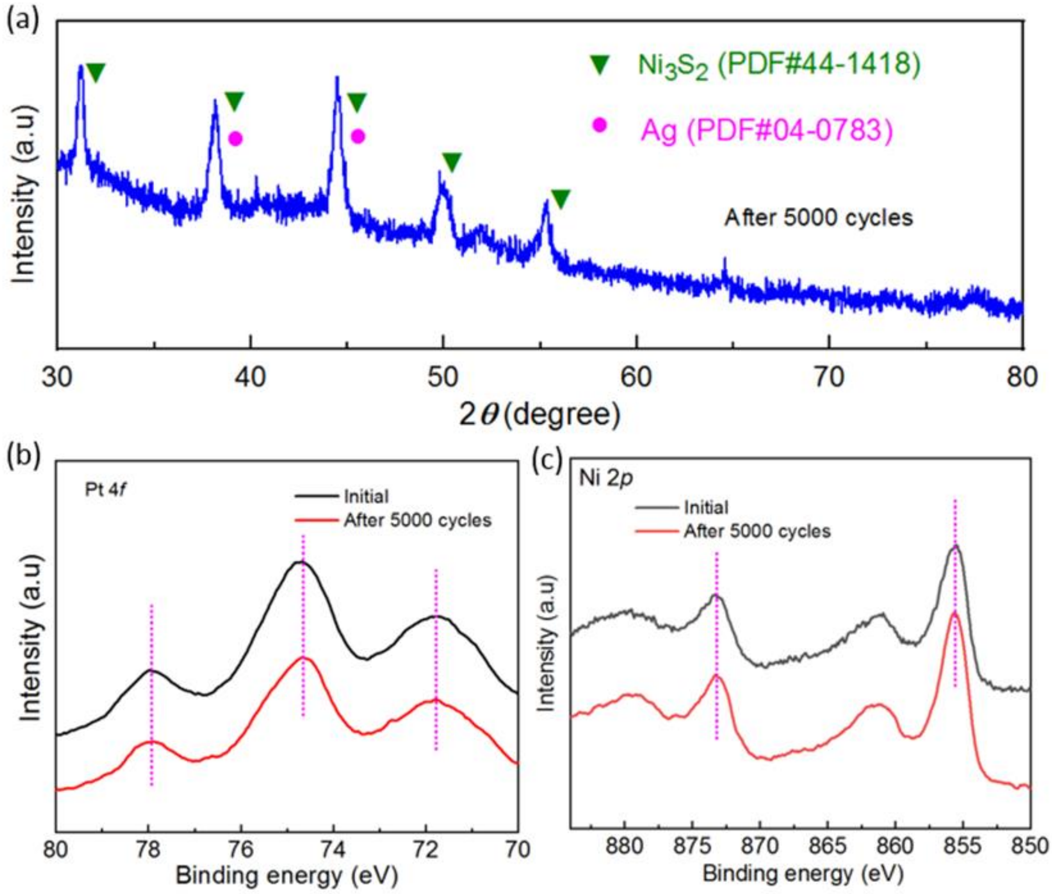

Figure S25. (a) XRD and (b-c) XPS data of Pt_{SA}-Ni_3S_2@Ag NWs after stability test.

Table S1. FT-EXAFS fitting results of Pt_{SA}-Ni_3S_2, and Pt foil was given as a reference.

	Shell	*CN*	R(Å)	σ^2	ΔE_0	R factor
Pt foil	Pt-Pt	12	2.76±0.01	0.004	10.4±0.6	0.0017
Pt_{SA}-Ni_3S_2	Pt-S	3.07±0.3	2.25±0.03	0.002	-6.4±4	0.0081
	Pt-Cl	2.1±0.3	2.02±0.04	0.006		

CN: coordination numbers of identical atoms; *R*: interatomic distance; σ^2: Debye-Waller factors; ΔE_0: energy shift. *R* factor: goodness of fit. S_0^2 was set to 0.74, according to the experimental EXAFS fit of Pt foil reference by fixing *CN* as the known crystallographic value.

Table S2. Comparison of HER performances for Pt_{SA}-Ni_3S_2@Ag NWs with other reported single-atom metal HER catalysts.

	Electrocatalysts	Bader charge value of metal atom (e)	Free energy of hydrogen (ΔG_{H^*}, eV)	Free energy of water (ΔG_{H2O^*}, eV)	Electrolyte	Overpotential at 10 mA cm^{-2} (mV)	Tafel slop (mV dec^{-1})	Reference
**1**	**Pt_{SA}-Ni_3S_2**	**0.017**	**-0.06**	**-0.67**	**1.0 M KOH**	**33**	**34.7**	**This work**
2	Pt_{SA}-C_1N_1	0.23	0.06	0.30	1.0 M KOH	46	36.8	Nat. Commun., **2020**, 11:1029
3	Pt_{SA}/m-WO_{3-x}	-	-	-	0.5 M H_2SO_4	38	45	Angew. Chem., Int. Ed., **2019**, 131: 16184.
4	Pt_{SA}/$Co_{0.85}$Se	-	-0.08	0.49	1.0 M PBS	55	35	Nat. Commun., **2019**, 10: 1743.
5	PtN_x/TiO_2	-	-0.13	-	0.5 M H_2SO_4	67	34	Nano Energy, **2020**, 73: 104739
6	Pt_{SA}-$Co(OH)_2$	0.47	-0.088	-0.764	1.0 M KOH	29	35.7	Energy Environ. Sci., 2020, 13:3082.
7	Pt_{SA}/S-C	-	-0.07	-	0.5 M H_2SO_4	53	46.92	Nat. Commun., **2019**, 10: 4977.
8	Ru_{SA}-N/S-$Ti_3C_2T_x$	-	0.08	-	0.5 M H_2SO_4	76	90	Adv. Mater. **2019**, 1903841
9	Pt@PCM	-	-	-	0.5 M H_2SO_4	105	63.7	Sci. Adv., **2018**, 4: 6657.

10	WS_2-Pt_{SA}	-	-	-	0.5 M H_2SO_4	40	27	Adv. Mater., **2017**, 1704779.
11	Ru_{SA}-MoS_2/CC	-	0.327	-	1.0 M KOH	41	114	Appl. Catal., B, **2019**, 249: 91.
12	Ru_{SA}-P_2@NPC	-	0.233	-	0.5 M H_2SO_4	38	38	Angew. Chem., Int. Ed., **2017**, 56: 11559.
13	Pt_{SA}/OLC	-	-	-	0.5 M H_2SO_4	38	36	Nat. Energy, **2019**, 4: 512.
14	Ru_{SA}/N-C NWs	-	-0.58	-	0.1 M H_2SO_4	47	14	Nat. Commun., **2019**, 10: 1.
15	Pt_{SA}-graphdiyne	-	-0.09	-	0.5 M H_2SO_4	65	46.6	Angew. Chem. Int. Ed. **2018,** 57: 9382.
16	$Mo_2TiC_2T_x$-Pt_{SA}	-	-0.19	-	0.5 M H_2SO_4	30	30	Nat. Catal., **2018**, 1: 985.

Supplementary Note 1. Turnover frequency (TOF) calculation of the Pt-based catalysts.

The TOF per Pt site of the fabricted Pt-based catalysts in this work was calculated by using the formula:

$$\textbf{TOF (H}_2\textbf{/s)} = \frac{\textbf{\# total hydrogen turnover per geometric area}}{\textbf{\# active sites per geometric area}}$$

The total number of hydrogen turnovers could be obtained by the current density as the following formula:

# total hydrogen turnover:

$$= \left(|j|\frac{\text{mA}}{\text{cm}^2}\right)\left(\frac{1\ \text{C/s}}{1000\ \text{mA}}\right)\left(\frac{1\ \text{mol e}^-}{96485.3\ \text{C}}\right)\left(\frac{1\ \text{mol}}{2\ \text{mol e}^-}\right)\left(\frac{6.022\times10^{23}\ \text{molecules H}_2}{1\ \text{mol H}_2}\right)$$

$$= 3.12\ \times\ 10^{15}\ \frac{\text{H}_2/s}{\text{cm}^2}\ \text{per}\ \frac{\text{mA}}{\text{cm}^2}$$

The number of catalytic active sites in the Pt-based catalysts were calculated according to the mass loading on the electrode, the Pt contents and the Pt atomic weight with a hypothesis that each Pt atom accounts for one catalytic active site:

# Pt active site:

$$=\left(\frac{\text{catalyst loading per geometric area }(x\ \text{g/cm}^2)\times\text{Pt wt\%}}{\text{Pt }M\text{w (g/mol)}}\right)\times\left(\frac{6.022\times10^{23}\text{Pt atoms}}{1\ \text{mol Pt}}\right)$$

Hence, the Pt active site in Pt_{SA}-Ni_3S_2 can be calculated to be:

$$= \left(\frac{0.024\times10^{-3}}{195.08\ \text{g/mol}}\right)\times\left(\frac{6.022\times10^{23}\text{Pt atoms}}{1\ \text{mol Pt}}\right)$$

$$= 7.4\ \times\ 10^{16}$$

So, the TOFs of per Pt site in Pt_{SA}-Ni_3S_2 can be calculated to be:

$$= \frac{3.12\times10^{15}}{7.4\times10^{16}}\ \times\ |j|$$

$$= 0.042\ \times\ |j|$$
